# An Overview of Engineered Hydrogel-Based Biomaterials for Improved *β*-Cell Survival and Insulin Secretion

**DOI:** 10.3389/fbioe.2021.662084

**Published:** 2021-08-26

**Authors:** Azin Ghasemi, Elham Akbari, Rana Imani

**Affiliations:** Department of Biomedical Engineering, Amirkabir University of Technology (Tehran Polytechnic), Tehran, Iran

**Keywords:** diabetes, hydrogels, insulin secretion, biomaterials, islet encapsulation

## Abstract

Islet transplantation provides a promising strategy in treating type 1 diabetes as an autoimmune disease, in which damaged β-cells are replaced with new islets in a minimally invasive procedure. Although islet transplantation avoids the complications associated with whole pancreas transplantations, its clinical applications maintain significant drawbacks, including long-term immunosuppression, a lack of compatible donors, and blood-mediated inflammatory responses. Biomaterial-assisted islet transplantation is an emerging technology that embeds desired cells into biomaterials, which are then directly transplanted into the patient, overcoming the aforementioned challenges. Among various biomaterials, hydrogels are the preferred biomaterial of choice in these transplants due to their ECM-like structure and tunable properties. This review aims to present a comprehensive overview of hydrogel-based biomaterials that are engineered for encapsulation of insulin-secreting cells, focusing on new hydrogel design and modification strategies to improve *β*-cell viability, decrease inflammatory responses, and enhance insulin secretion. We will discuss the current status of clinical studies using therapeutic bioengineering hydrogels in insulin release and prospective approaches.

## Introduction

Type 1 diabetes mellitus (T1DM) is a chronic metabolic disease that is characterized by the production of insufficient or no insulin as a result of the destruction of *β*-cells (Y. J. Lin et al., 2019), which can result in hyperglycemia, hypoglycemic unawareness, and ketoacidosis (Omami et al., 2017). According to a recent WHO report on diabetes published in 2020, there are more than 420 million people living with diabetes, and it is the seventh leading cause of death and a significant cause of heart attacks, strokes, kidney failure, and blindness. International Diabetes Federation estimates this number could reach over 592 million by 2035 (Guariguata et al., 2014; Baghban; Taraghdari et al., 2019). The conventional therapeutic approach in treating T1DM is exogenous insulin replacement therapy, which requires continuous subcutaneous injections of insulin. Although insulin injection effectively controls blood glucose levels, it cannot recapitulate the physiological pancreatic insulin secretion pattern and thus may result in complications such as neuropathy, nephropathy, retinopathy, and heart disease (Zamboni and Collins, 2017; Shrestha and Regmi, 2020).

Pancreatic islet transplantation is an alternative and less-invasive therapeutic method in pancreatic *β-*cell replacement. This procedure was first performed in 1974 but did not produce a successful outcome until 2000, when Shapiro et al. have presented an optimized protocol for T1DM treatment (Shapiro et al., 2000). This protocol, universally known as “Edmonton protocol,” involves the extraction (mechanical and enzymatic) of islets from a deceased donor’s pancreas, followed by the infusion of donor islets through the portal vein of patients’ liver. In this treatment, patient recovery is quick and the risk of infection is minimal. However, transplantation rejection by the host immune response and an overall lack of islet donors are major caveats of the protocol (Dinnyes et al., 2020). Numerous studies have demonstrated that patients treated by the Edmonton protocol had an insulin-independent rate of approximately 50% 5 years after transplantation. These results also reported that the extraction process encounters mechanical, physiological, and particularly immunological hurdles, limiting *β*-cell functionality post-transplantation. The reasons for unsuccessful long-term grafts could be categorized into two main classes (Qi, 2014): immunosuppression and non-immunosuppressive-associated factors. While the administration of immunosuppressive medications prevents allogeneic rejections associated with transplantation in the long term, even the most advanced forms of these medications may have toxic effects on recently transplanted cells and may lead to the dysfunction of other vital organs. Furthermore, the initiation of recurrent autoimmune responses may destroy the transplanted cells. In the latter category, the gradual dysfunction of islets may be caused by non-immunosuppressive factors such as insufficient islet mass, poor islet quality, instant blood-mediated inflammatory reaction (IBMIR), and hypoxia-related islet cell death. The last two factors were observed after transplantation in the liver through the portal system (Carlsson et al., 2001; Hilbrands et al., 2009).

Protection of the transplanted secretory cells against the recipients’ immune systems can mitigate the non-desirable limitation of transplantation and increase the efficacy of such treatment. Namely, the microencapsulation of *β*-cells through biomaterials has been suggested as an immunomodulatory approach.

Among various biomaterials, hydrogels, three-dimensional polymeric networks with high water content and crosslinked nature are strategically introduced to encapsulate biological elements and cells (Espona-Noguera et al., 2019). Current engineering approaches are designed to improve the outcomes of islet transplantation using engineered, hydrogel-based encapsulation devices, aiming to protect *β*-cells against host immune responses and facilitate the exchange of vital molecules such as oxygen and nutrition.

Different synthesis approaches in macro-, micro-, and nanoscales are used to prepare hydrogels for cell encapsulation. Macroscale fabrication involves preparing injectable and pre-synthesized hydrogels that can mimic the pancreas extracellular matrix (ECM). Micro- and nanodevices generally include micro- and nanoparticles in which matrix structure endows a 3D aggregation or station of *β*-cells for cell delivery (Dimitrioglou et al., 2019). Engineered hydrogel-based biomaterials provide a niche for the protection of encapsulated *β*-cells against the immune system and increase their survival *in vivo* (Crisóstomo et al., 2019). These hydrogels can be functionalized with bioactive ligands, which are primarily selected from the main components of the islet native ECM microenvironment. Such bioactive hydrogels can favor the function of insulin-producing cells due to the natural responsibility of ECM in interactions with cells, which induces cell survival, migration, morphology, and differentiation (Beenken-Rothkopf et al., 2013).

The aforementioned hydrogel-based approaches could be much more effective when associated with vasculogenesis strategies. Although each islet has an average capacity for insulin secretion, the lack of angiogenesis reduces this capacity due to the pancreatic islets’ highly vascularized nature. This vascularization supports blood glucose sensitivity in order to secrete insulin (Weaver et al., 2017). Alteration in the mechanism of islets vascularization induces islet dysfunction (Brissova et al., 2006). In this case, some growth factors and cell resources can be co-transplanted by islets and stimulate capillary formation.

This review aims to provide an update on hydrogel-based approaches for T1DM treatment. First, we introduce different methods of hydrogel fabrication adopted for islets or *β*-cell encapsulation. Then, biocompatibility and vascularization strategies, which are applied to these engineered hydrogel-based materials to improve cells’ viability, and insulin secretion are comprehensively discussed.

## Hydrogel-Based Encapsulation Devices

Cell encapsulation was first described by Chang, 1964 and then applied by Lim (Lim and Sun, 1980) for the treatment of diabetes. Lim’s study showed that the encapsulation technique enhanced the survival of the islet from 8 days to 3 weeks (Desai and Shea, 2017). As mentioned, protecting the cells against immune systems is considered one of the most significant challenges in islet transplantation (Omami et al., 2017). One of the primary goals of islet encapsulation is to suppress the interaction of the immune cells within transplantation sites and ensure the proper transfer of oxygen, growth factors, hormones, and nutrients (Bünger et al., 2005). Using biocompatible encapsulation devices, consisting of semi-permeable membranes with sufficient nutrition, oxygen, and insulin transfer, can protect *β*-cells against the invasion of the host immune cells without requiring immunosuppression (Dhamecha et al., 2019a).

In recent years, encapsulation technologies demonstrated desirable applications in encapsulation of islets mainly due to their ability to exclude the effect of the immune system, such as antibodies and immune cells (Caserto et al., 2020). An encapsulation device can contain one or several islets and its size can vary from nano- to macroscale. These encapsulation devices are mainly classified into three types: macrodevices, microdevices, and nanodevices.

Macrodevices, divided into “intravascular” and “extravascular,” are diffusion chambers or hydrogels of different shapes containing a large number of islets (Primavera et al., 2020). The low diffusibility of oxygen and nutrients through the thick membrane of macrodevices has resulted in the emergence of micro- and nanoencapsulation technologies. While the thinner membrane addresses the oxygen and nutrition diffusion in microdevices, biocompatibility remains a significant challenge. One of the challenges regarding micro- and macroencapsulation of *β*-cells is the probability of fibrotic tissue formation around transplantation sites. In addition to the surface modification of encapsulating materials, some findings have revealed that the size of encapsulation systems can also suppress immunological responses and avoid the formation of a thick layer of fibrotic tissue around the transplanted device (Ernst et al., 2019; Kwiatkowski et al., 2020). Thus, the use of nanotechnology in the encapsulation process has gained significant attention.

Today, various types of inert biomaterials such as alginate, agarose, and polyethylene glycol (PEG), in the form of a hydrogel, are used to encapsulate islets (Omami et al., 2017). Zamboni et al. have categorized hydrogels in three major groups, based on their crosslinking and molecular interactions: 1) first-generation hydrogels, a group of hydrogels crosslinked by covalent interactions, 2) second-generation hydrogels, crosslinked via non-covalent interactions such as hydrophobic and electrostatic interactions, and 3) third-generation hydrogels, formed via interpenetrating polymer networks consisting of two or more networks of different components. First- and second-generation hydrogels are primarily used in islet encapsulation (Zamboni and Collins, 2017), including *in situ* forming, responsive, and smart hydrogels.

Given the importance of encapsulation device size, hydrogel-based encapsulation carriers can be categorized by their sizes as follows: macro-, micro-, and nanodevices.

### Macrodevices

#### Injectable Hydrogels

One form of hydrogels used in islet encapsulation is injectable hydrogels. Injectable hydrogels possess fluidic characteristics, which can be injected or sprayed on the implantation site and formed *in situ*. The preference of such hydrogels over pre-synthesized, pre-formed solid hydrogels is their non-invasive administration and their capacity to the steric of host tissues (Sanandiya et al., 2019). Different methods, including non-toxic chemical cross-linkers, enzymatic crosslinking, physical interactions (e.g., hydrophobic and ionic interactions), and supramolecular chemistry, are adapted to prepare the injectable hydrogels (Yang and Hahn, 2014)**.**

In numerous studies, single islet cells are artificially re-aggregated after dispersion in culture media and seeding in concave molds. Cells aggregate and form a spheroid structure after seeding for a certain period. The spheroid structures are then encapsulated within hydrogels for immune protection. Islet spheroids are fragile and tend to disaggregate; consequently, handling and encapsulation in hydrogel-based devices are time-consuming, with the possibility of cell damage. Furthermore, reproducing the encapsulation process on a large-scale basis is a challenging issue. To tackle these hurdles, injectable hydrogels provide a single platform where both spheroid formation and encapsulation can be performed sequentially.

Various studies have investigated injectable hydrogels for islet encapsulation. Haque et al. have used injectable Matrigel^®^, a commercial thermosensitive ECM-derived hydrogel, as a carrier for the local delivery of *β*-cells and liposomal clodronate, which is a novel agent that could improve the survival time of *β*-cells (Haque et al., 2014). Matrigel^®^, at its jelly-like state, was added to *β*-cells’ suspension to prepare the delivery system, and then it was subcutaneously injected into the scruff of knock-out mice. The results indicated the injectable Matrigel^®^ and co-encapsulation of liposomal clodronate presents a successful strategy toward improving anti-inflammation and immunoprotection of locally delivered islets for T1DM treatment. Furthermore, they showed that this cell delivery system increased islet survival from 10 to 60 days significantly (Schaschkow et al., 2020).

Alginate is a naturally occurring, biocompatible, and cost-effective polysaccharide with mild gelation properties. These desirable properties and tunable rheological and physicochemical properties make it a functional biomaterial in fabricating injectable hydrogels used for *β*-cell encapsulation (Edward, 2009). In a study conducted by Noguera et al., an injectable alginate-based hydrogel with versatile physicochemical properties was utilized for *β*-cell transplantation. The hydrogel was prepared by ionic crosslinking, and the gelation time was retarded using Na_2_HPO_4_ as a retarding agent. Increasing gelation time of *in situ* forming hydrogel is advantageous because it improves flexibility and handling, which are especially useful in clinical applications. The results showed that the use of Na_2_HPO_4_ had a significant impact on delaying hydrogel formation at the implantation site and rectifying hydrogel’s mechanical and physiological properties, which improved *β*-cells’ biocompatibility and growth (Espona-Noguera et al., 2018).

**FIGURE 1:**
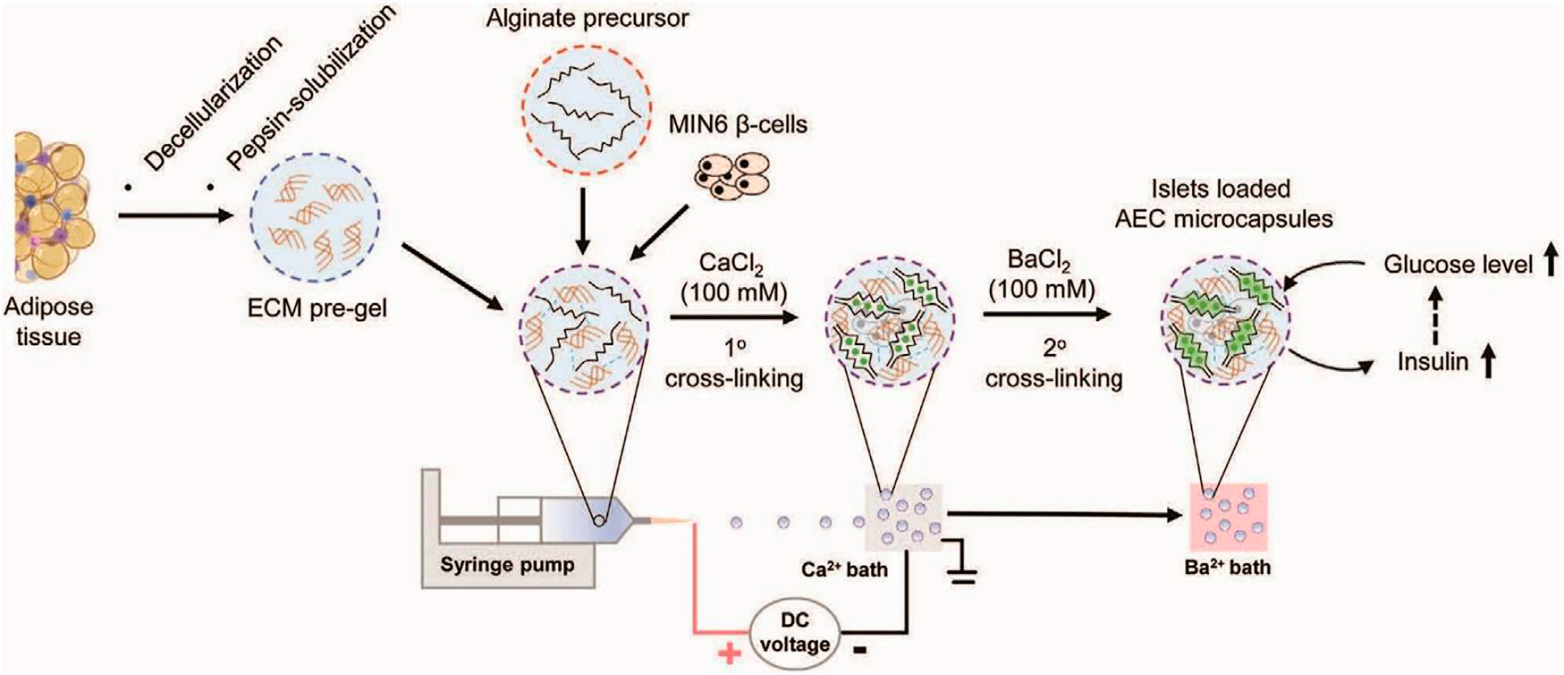
Schematic illustrating fabrication steps alginate microcapsules containing human adipose tissue-derived ECM and microencapsulation of pancreatic islets (J. K. Wang J. K. et al., 2020).

In another study reported by Wang et al., novel thermosensitive interpenetrating networks (IPN) of alginate and human adipose tissue-derived ECM were fabricated as a biomimetic encapsulate environment for islets delivery (J. K. Wang J. K. et al., 2020). For encapsulation, islets were added to an alginate solution, then the system was crosslinked through ionic gelation, and finally, the microencapsulated structure was added to the ECM-derived hydrogel (Figure 1). *In vitro* cellular studies showed that the system was biocompatible and tolerated by immunity. The cells’ population was increased seven times over the course of a week compared to non-encapsulated cells.

PEG-based synthetic hydrogels are regarded as one of the most promising carriers in cell delivery applications. This is due to their intrinsic low protein adsorption, immunoprotective properties, minimal inflammatory invasion, and exceptional biocompatibility (Bai et al., 2020). In a study, Knobeloch et al. have developed an injectable PEG hydrogel that supports the survival of islet cells *in vitro* and *in vivo* (Knobeloch et al., 2017). The hydrogel was formed via Michael-type addition of a multi-arm PEG-vinyl sulfone (VS) and a PEG-based dithiol cross-linker. Islets were encapsulated in the hydrogel by gently pipetting hydrogel solution to the cultured cells. About 90 s before the complete gelation of the hydrogel, the solution was injected on the transplantation site. With almost 100 islets encapsulated in the hydrogel, monitoring the blood glucose levels showed a significant reduction, from 600 to 200 mg/ml in about 2 days post-implantation. In a similar condition, the non-encapsulated islets reduced blood glucose level from 570 to 470 mg/ml (Lin et al., 2011).

Recently, Schaschkow et al. have fabricated a blood plasma-based environment for islets encapsulation. In this study, plasma was mixed with the viscosity-enhancer hydroxypropyl methylcellulose (HPMC) to attain an injectable hydrogel. Hydrogel formation in an *in vivo* environment was due to the impact of the circulating thrombin, which endows the rapid polymerization of the fibrinogen content of plasma into fibrin gel. After the characterization of the hydrogel and encapsulated islets, the delivery system was injected into the omentum, which is considered an alternative recipient site for islet transplantation (Schaschkow et al., 2020). The SEM observation of encapsulated islets in *in vitro* showed that the fibrin existing in the plasma could be polymerized and formed a nest for encapsulated islets and increased their survival. A reduction by at least a fourth of the original needs of islets (non-encapsulated ones) revealed the practical and cost-effective application of this system in controlling glucose concentration for diabetic therapy.

#### Pre-Synthesized, ECM-Liked Scaffolds

Pre-synthesized, ECM-like scaffolds are hydrogel-based macrostructures used as a niche for islets in which they are placed and then transplanted into the omentum of patients with type 1 diabetes. These systems endow long-term insulin independence after transplantation for diabetic patients (Ravnic et al., 2017). The ECM of islet cells comprises collagens I and III-VI, fibronectin, and laminin and any incorporation of these materials in the synthetic ECM structures would considerably enhance *β*-cells’ functionality. For having high metabolic activity, *β*-cells should receive high nutrition and oxygen. Thus, fabrication of engineering macrosystems transferring enough nutrition and oxygen to the cells is of great importance for designing this kind of system (Gurlin et al., 2020).

Although a wide array of engineering techniques is adopted in order to fabricate pre-synthesized ECM-liked scaffolds, in this review, we categorized these techniques into four primary groups: 1) 3D bioprinting, 2) tissue decellularization, 3) self-assembling peptides,and 4) synthetically derived chemical and physical hydrogels. Some research studies are discussed in detail as follows:

• 3D bioprinting

3D bioprinting, as an additive manufacturing technique, is based on the deposition of biomaterials with cells during the fabrication process or loaded by cells later on. The positioning biomaterials and viable cells in a stacking layer by layer at a desirable position, 3D bioprinting emerged as an applicable technique for cell encapsulation for different biomedical purposes (Lee et al., 2018). A combination of biomaterials called “bioink” is used as the printing precursor in these systems. Based on the methods of functionality, these devices are categorized into four main groups: extrusion-based, inkjet, stereolithography-based, and laser-assisted bioprinting methods (Kengla et al., 2017; Derakhshanfar et al., 2018). Among these four groups, inkjet and extrusion-based bioprinting techniques apply to islet cell encapsulation. The functionality of each method and its advantages and disadvantages have been previously discussed in detail in different studies (Kengla et al., 2017; Ravnic et al., 2017; Derakhshanfar et al., 2018; Lee et al., 2018; Leberfinger et al., 2019) and are summarized in Table 1.

**TABLE 1:** A different methods of 3D bioprinting, along with their corresponding advantages and disadvantages, utilized for islet encapsulation.

Bioprinting technique	Mechanism of function	Advantages and drawbacks	Examples of bioinks used for islets encapsulation	Schematic illustration	References
**Inkjet bioprinting**	Piezoelectric or thermal-driven mechanisms	Adv.: printing low viscosity biomaterials, low cost, high resolution	Gelatin, alginate	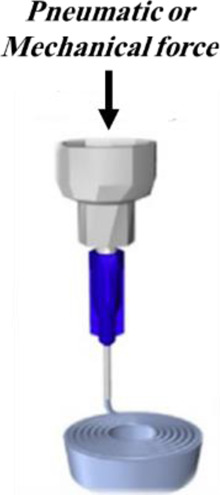	Wang, et al., 2017b; Llacua et al., 2018; Smink and Paul de Vos 2018; Townsend and Gannon 2019; Mridha et al., 2020); Hu et al., 2021
		Disadv.: low cell density, not applicable for vertical structures			
**Extrusion-based bioprinting**	Pneumatic, mechanical or solenoid driven mechanism	Adv.: High cell density, wide range of printable biomaterials	Alginate, methylcellulose, PCL, gelatin	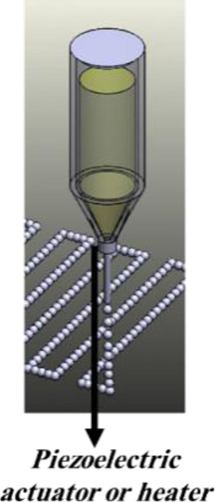	(Peloso et al., 2018; Smink and Paul de Vos., 2018; Duin et al., 2019; aewook; Kim et al., 2019b; Y.; Zhu et al., 2020)
		Disadv.: low resolution and speed			

Duin et al. have developed a 3D scaffold produced by an extrusion-based bioprinter whose bioinks are composed of 3% alginate, 9% methylcellulose, and an appropriate number of cells encapsulated in the hydrogel medium. MTT, DTZ staining assays, and immunofluorescence staining assay were used for the characterization of survival. The glucose-stimulated insulin secretion (GSIR) was used to analyze islets’ responses to the glucose. The results revealed that the size of alginate-encapsulated islets was significantly bigger than alginate/methylcellulose encapsulated cells, while there was a significant reduction in the size of alginate-encapsulated cells from days 4 to 7 of culture. Semi-quantitative assessment of cell viability in 7 days also expressed an insignificant difference between alginate/methylcellulose-encapsulated and alginate-encapsulated cells where alginate/methyl cellulose-encapsulated cells had almost 80% of viability. Qualitative analysis of TUNEL and DAPI stained islets showed apoptosis of cells cultured in the outer area of the scaffold. On the other hand, for the non-encapsulated cells, apoptosis occurred not only in the outer layer of cells but also in the center part. Functionality assessments also revealed that the alginate/methylcellulose blend scaffold had a higher capacity of insulin uptake due to this hydrogel’s higher microporosity (Duin et al., 2019).

• Tissue decellularization

Tissue decellularization is a technique in which cellular components are stripped from tissue or solid organ, and an acellular 3D structure is composed of ECM. As mentioned, pancreas ECM that comprises collagen, laminin, fibronectin, and fibrin is involved in cytoskeletal remodeling, contractility, and cells differentiation. The most abundant ECM molecules are collagen types IV and VI and laminins (e.g., laminin-332 and laminin-511) (Llacua et al., 2018; Peloso et al., 2018). However, it should be considered that insufficient concentration of some molecules can have a detrimental effect on cells’ viability and function, and a high concentration of collagen IV hampers islets function (Smink and Paul de Vos, 2018; Townsend and Gannon, 2019). The most significant advantages of a decellularized pancreas ECM are its biocompatibility and bioactivity, which could increase islet survival, reduce cytotoxicity, and consequently improve islets’ function.

Decellularized pancreas matrix or its modified structures as 3D scaffolds used a niche for *β*-cell encapsulation. For instance, in a recent study by Zhu et al., electrospinning hybrid scaffolds with silk fibroin (SF) and pig pancreatic decellularized ECM (P-dECM) were fabricated for *β*-cell encapsulation. The ECM-derived powder was mixed with silk fibroin solution at a specific ratio, and the final solution was electrospun by self-assembled electrospinning equipment. To study the impact of ECM components on cell functionality, the viability and insulin secretion ability of cells were compared with non-encapsulated cells. *In vitro* cytotoxicity assay indicated no significant difference in cell viability between non-encapsulated and encapsulated cells. *In vitro* insulin release capacity of encapsulated islets cells was evaluated by GSIS assay. Results showed that under high stimulation of glucose, the amount of insulin secretion from encapsulated cells was significantly higher than that from non-encapsulated ones (Y. Zhu et al., 2020). Incorporation of decellularized ECM into inert hydrogel substrate (e.g., alginate) not only may mimic the native pancreas ECM and enhance encapsulated islets function but also can improve the mechanical stability of hydrogels (Enck et al., 2021).

Kim et al. have used a P-dECM as a bioink for an extrusion-based 3D bioprinting of islets or *β*-cells. To investigate the effect of ECM components on cells’ viability and functionality, they have used immunofluorescence staining and GSIS, respectively. A quantitative cell viability study showed no significant difference between encapsulated and non-encapsulated cells, and islets were maintained up to 5 days at over 60% of viability. Insulin release of the encapsulated islets compared to islets encapsulated in alginate and collagen hydrogel revealed that the GSIS index of the P-dECM system was about 1.346 higher than the alginate and collagen system. The results demonstrated the influential role of the ECM components on cells’ viability and function of islets (Jaewook Kim et al., 2019a).

In another study, Jiang et al. have used the decellularized matrix of basement membrane-rich lung and bladder tissue of pigs as an *in situ* forming hydrogel. To encapsulate islets, cells were gently mixed with decellularized ECM pre-gel solution, and the islet-encapsulated hydrogel matrix formed at physiological condition (37°C and pH = 7.4) after 30 min. To investigate the effect of the ECM hydrogel on islet function, the GSIR from islets was evaluated using a dynamic perfusion system. *In vitro* assessment of the islets functionality was extended to 1 week post-encapsulation. Its results showed that the insulin secretion from the ECM hydrogel was four indexes higher than that from non-encapsulated cells. *In vivo* islets viability was assessed by visually characterizing cells via viability staining and confocal microscopy. Cells’ viability was monitored over 80 days and the results showed stability in ECM gels with no significant apoptosis and aggregation in this period (Jiang et al., 2019).

• Using self-assembling peptides

A self-assembling hydrogel is an extended form of nanofiber based on self-assembling peptides with a highly entangled network for biomedical applications. The pH, ionic strength, temperature, enzyme catalysis, and selective irradiation are stimuli employed to trigger the formation of these hydrogels (Banerjee, Radvar, and Azevedo, 2018). To assemble these systems, different sequences of peptides or hybrid peptides are adopted. A wide array of bioactive motifs with the source of natural amino acids has been introduced to the pure self-assembling peptides. Hybrid peptides include biocompatible biopolymers such as ECM mimic motifs like collagens, fibronectin, and polysaccharides such as hyaluronic acid (HA) (Lu and Wang, 2018). A self-assembling peptide-based hydrogel is used to enhance islets functionality and survival. Although *in vivo* evaluation of islets encapsulated in these systems is still challenging, the *in vitro* evaluation showed a significant increase in their functionality and viability (Xu et al., 2019).

To encapsulate *β*-cells in a hydrogel-based structure, Liu et al. have used a hybrid peptide sequence containing collagen IV and fibronectin motifs, and cell encapsulation in this hydrogel caused a 1 µU/ml increase in insulin secretion compared to that in the non-encapsulated cells (J. Liu et al., 2015). In another study, Huanget al. have fabricated a peptide amphiphile-based nanomatrix hydrogel to encapsulate islets. GSIS showed that cells’ encapsulation almost increased 8,477.78 µIU in glucose-responsive value when exposed to high glucose concentration (Huang et al., 2017).

• Other pre-fabricated chemical and physical hydrogels

In addition to the methods discussed above, different physical and chemical hydrogel fabrication methods are used for islet cell encapsulation. Some studies have tried the idea of using pre-fabricated hydrogels. In such studies, non-toxic microenvironments have been adopted as 3D engineering niches for the seeding of islets. This microenvironment also endows a platform for *β*-cell encapsulation to recreate the native physiological milieu (Peloso et al., 2018). In this regard, a wide array of hydrogel preparation techniques such as photopolymerization (Pratik K. Mutha, Robert L. Sainburg, 2008), ionotropic gelation, and chemical modification (Harrington et al., 2017; Crisóstomo et al., 2019) is adopted for *β*-cell encapsulation.

In these studies, modified hydrogels considerably increased islet cell survival and functionality. The impacts of engineering approaches in improving islets functionality are discussed in detail in *Current Strategies of Engineered Hydrogels for the Improvement of Insulin Secretion*.

### Microdevices

#### Microparticles

The most common microdevices among different encapsulation devices are microparticles broadly investigated and used in several islet encapsulation studies (Espona-Noguera et al., 2019). Microcapsules are often fabricated by microfabrication techniques such as batch emulsion and microfluidic devices. In this technique, an individual or small cluster of islets is surrounded by semi-permeable polymeric membranes, which facilitate nutrients and oxygen transfer and enable direct injection of *β*-cells into bloodstreams (Dhamecha et al., 2019a; Dimitrioglou et al., 2019; Daly et al., 2020).

**FIGURE 2:**
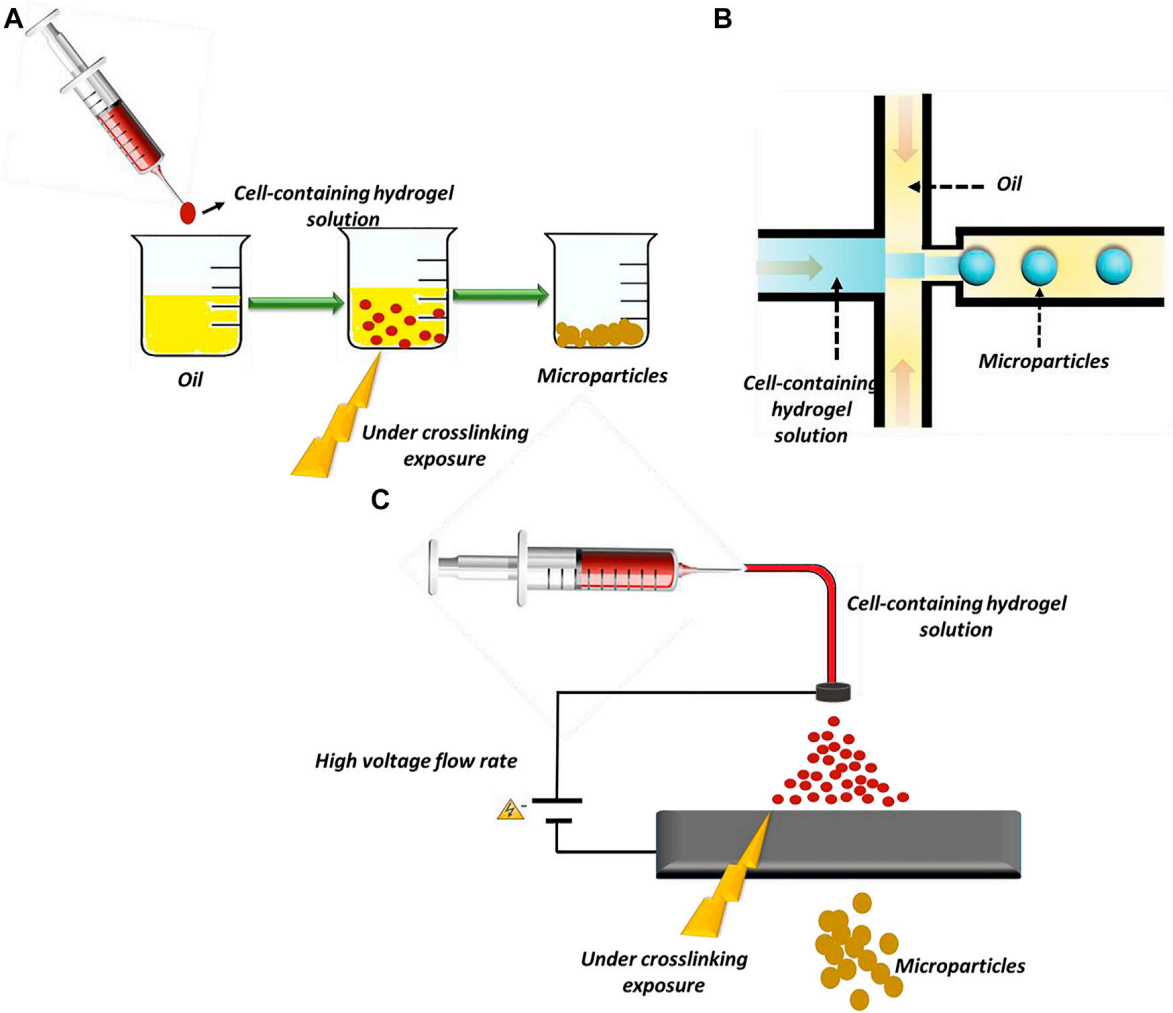
Methods of microparticles fabrication: **(A)** batch emulsion technique, **(B)** microfluidic emulsion technique, and **(C)** EHD spraying.

Daly et al. have fabricated hydrogel-based microparticles in five major groups: 1) batch emulsions, 2) microfluidic emulsions, 3) lithography, 4) electrohydrodynamic (EHD) spraying, and 5) mechanical fragmentation (Daly et al., 2020). Among these methods, batch emulations, microfluidic emulsions, and EHD spraying are widely used in *β*-cell encapsulation (Figure 2).

In the batch emulsion technique, microparticles form as a result of the pre-polymer crosslinking in an aqueous solution. In this straightforward method, the pre-polymer is solved in an aqueous solution and mixed with an oily phase. It is exposed to an external crosslinker source, and finally, microparticles form in the oily phase. The main limitation of this method is the absence of control over particles’ size and polydispersity (Ding et al., 2019; Daly et al., 2020). The microfluidic emulsion technique is a promising technique for particle preparation that provides reasonable control over particle size and dispersity (J. Wang et al., 2017a). In this technique, cells are incorporated in an aqueous phase, and the solution is pumped at a defined flow rate in a channel. The solution then joins an oily phase flowing from other channels. In these devices, shear forces and hydrophobic interactions induce cell-encapsulated microparticle formation (Wen et al., 2018; Daly et al., 2020).

In EHD spraying, microparticles are produced by extruding a hydrogel solution through a syringe while a voltage is applied at the needle tip (Daly et al., 2020). This method produces microparticles in a high throughput manner. In some studies, alginate microparticles, as the most prevalent biomaterials used for *β*-cells’ encapsulation, are produced by EHD spraying (Jeyhani et al., 2018; Correia et al., 2019).

As previously discussed, alginate microparticles are extensively used for *β*-cell encapsulation. Bochenek et al. have prepared chemically modified alginate microparticles by electrospraying method. This study showed chemically modified alginate microparticles with three triazole rings containing small molecules, Z1-Y15-Ba^2+^, Z2-Y12-Ba^2+^, and Z1-Y19-Ba^2+^. Then, the efficacy of prepared formulation in insulin release was assessed in non-human primate models *in vivo*. The microparticles were implanted laparoscopically and insulin secretion of the islets retrieved from the animal models was quantified by human insulin ELISA kit. The viability of post-isolation, post-encapsulation, and post-retrieval cells was also assessed using dual live/dead fluorescence staining. The results showed that chemical modification of alginate microparticles reduced macrophage activation effectively and the recruitment of myofibroblasts. Cell viability of modified microcapsules was also 93.5 and 90.0% after 1 and 4 months, respectively.

On the other hand, non-modified and non-encapsulated *β*-cells were identified by immune cells after a short period and excluded. They also provoked macrophage activation and resulted in fibrosis tissue formation. The high cell viability guarantees high glucose secretion, and the results of insulin secretion showed that almost 20 µU/ml per 10 islets was secreted in 4 months. It is noteworthy that insulin secretion was significantly higher in the presence of high glucose concentration (Bochenek et al., 2018).

In a recent study reported by Montanucci et al., alginate microcapsules were prepared by a micro-droplet generator with high voltage electricity. The microcapsules were used to co-aggregate postpartum umbilical cord Wharton’s Jelly–derived adult mesenchymal stem cells (MSCs). The encapsulation system was used as an immunoregulator containing human pancreatic islet-derived progenitor cells. The primary purpose of this study was to couple immunoregulatory activities of postpartum umbilical cord Wharton’s Jelly–derived adult MSCs with tracer insulin production by human pancreatic islet-derived progenitor cells. The particular microenvironment offered by alginate-based microcapsules was adopted to arrest the progression of T1DM. Mimicking ECM-like matrix, alginate microcapsules promoted the formation of 3D cell co-aggregation because the formation of cell spheroids is essential for the cell re-differentiation process. For *in vivo* studies, microcapsules were grafted intraperitoneally into knock-out mice, and results of insulin secretion from the retrieved cells were expressed as the percentage of fractional insulin release out of total insulin content. Blood glucose levels were measured and followed up throughout 180 days, and results indicated that after 2 weeks of implantation, blood glucose levels dropped from 300 to 500 mg/day to below 200 mg/day. The study demonstrated the remarkable role of the ECM-like alginate microcapsule and the co-encapsulation of postpartum umbilical cord Wharton’s Jelly–derived adult MSCs in protecting insulin-producing cells from the host immune system. It also showed that transplantation of human pancreatic islet-derived progenitor cells alone worked within a specific time frame, while the prepared microsystem extended graft function for 180 days (Montanucci et al., 2020).

In another research, Lew et al. have studied the potential application of porcine xenograft *β*-cells encapsulated in alginate microcapsules for transplantation. The encapsulated cells were co-cultured with exenatide-loaded poly(lactic-co-glycolic acid) (PLGA) microspheres. Exenatide is a glucagon-like peptide-1 receptor agonist that works by increasing insulin release from the pancreas and decreases excessive glucagon release. PLGA and alginate microspheres were prepared using the precision particle fabrication method, an acoustic-based spraying method. *In vitro* viability and function of *β*-cells were assessed by the Trypan Blue exclusion and insulin ELISA assay, respectively. After 21 days of encapsulation, the viability of *β*-cells co-encapsulated with exenatide-loaded PLGA microparticles was 71.1%, while it was about 45.1% for non-encapsulated cells. To evaluate the effectiveness of the prepared delivery system, *β*-cells’ functionality was investigated. The decreasing amount of insulin produced by non-encapsulated *β*-cells after 3 days indicated *β*-cells’ death. On the other hand, the result of the function of encapsulated cells expressed a high amount of insulin secretion during the entire 21 days (Lew et al., 2018).

**FIGURE 3:**
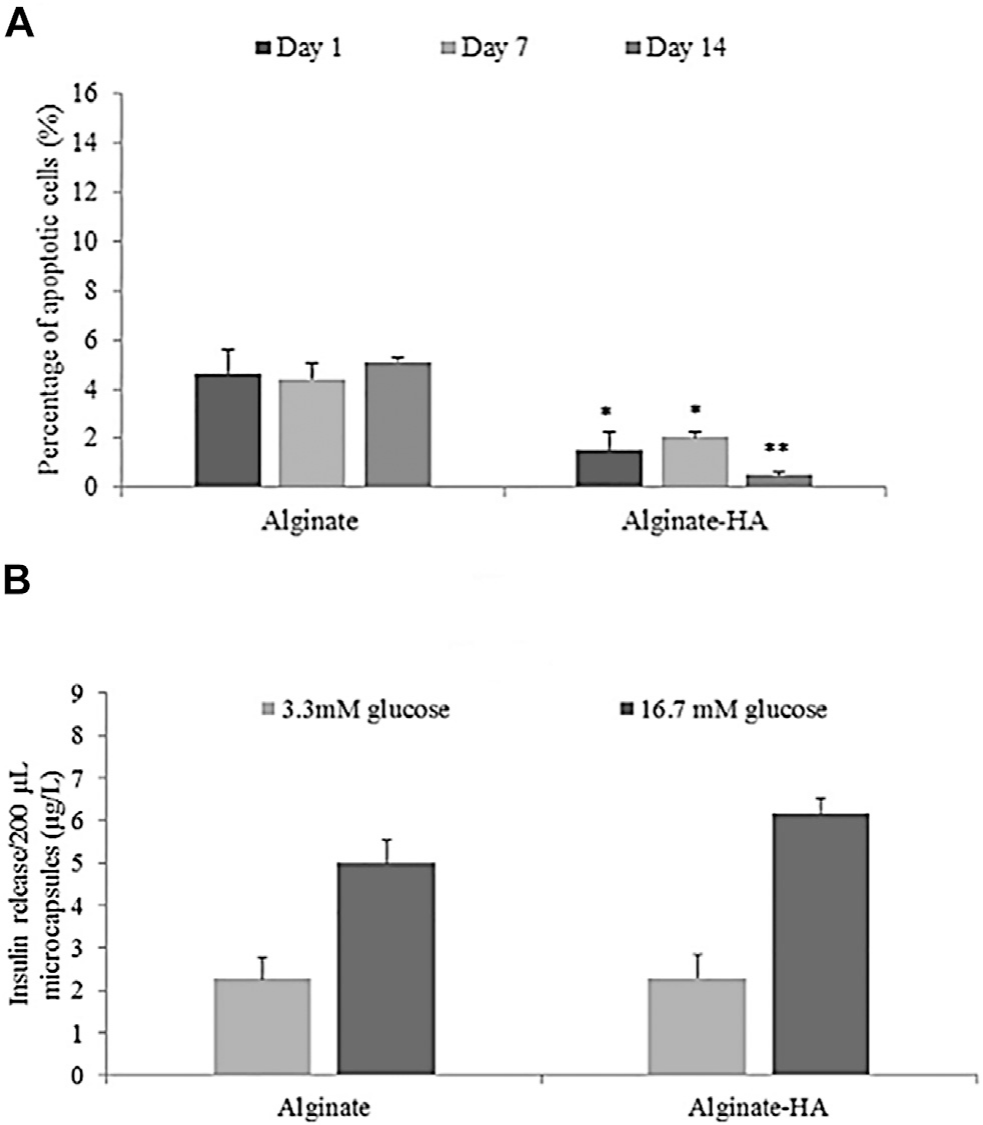
Cell viability **(A)** and insulin secretion **(B)** from hyaluronic acid-alginate hybrid microparticles encapsulating islets (Cañibano-Hernández et al., 2019).

In another study, Cañibano-Hernández et al. have studied a HA-alginate hybrid microcapsule to enhance the viability and function of *β*-cells. It has been reported that as one of the significant components of pancreatic ECM, HA can improve alginate microcapsules’ ability to mimic pancreatic ECM. The microparticles are prepared using an electrostatic atomization generator, which is an EHD spraying method. As shown in Figure 3, the viability of *β*-cells encapsulated in HA-alginate microcapsules was significantly higher than that of non-encapsulated cells after 14 days of encapsulation. However, results showed that HA inclusion did not alter cells’ ability in insulin secretion (Cañibano-Hernández et al., 2019).

Weaver et al. have compared two microencapsulation devices for encapsulation of *β*-cells: alginate microcapsules prepared by an EDH spraying device and PEG microcapsules prepared by a flow-focused microfluidic device. The purpose of this study was to demonstrate the priority of PEG-tripeptides, Arg-Gly-Asp (RGD), based microcapsules over alginate microcapsules in insulin secretion and adaptability for transplantation at vascular tissues. For transplantation, microparticles were dispersed in a vasculogenic PEG-based hydrogel whose monomer was functionalized with VEGF growth factor and RGD peptides. Microparticles were then injected at the epididymal fat pad. Results expressed a rapid rejection of non-encapsulated islet grafts within 2 weeks. Moreover, all alginate microcapsules escaped from the transplantation site to the intraperitoneal cavity within 2 weeks after transplantation. On the other hand, PEG-RGD-encapsulated microcapsules exhibited consistent localization at the transplantation site. Results of insulin secretion showed that alginate-encapsulated islets had a significantly lower insulin release than PEG-RGD-encapsulated cells. This is due to the islet loss during the encapsulation process, as the electrostatic encapsulation in alginate microparticles resulted in a more significant loss of islets (Weaver et al., 2018).

In a similar study presented by Laporte et al., *β*-cell-encapsulated alginate microcapsules were included by MSCs and RGD peptides. The primary purpose of including RGD and co-culturing of MSCs was to improve their biocompatibility, increase *β*-cell functionality, and prevent fibrosis overgrowth around the capsule. The microparticles were fabricated by a microfluidic device, and their functionality was evaluated. Results expressed an increase in *β*-cell viability when they were co-cultured with MSCs. On the other hand, insulin secretion was significantly higher in MSCs and RGD-containing encapsulated cells than in the two other samples. Also, results showed that insulin secretion increases 2.5% in those microcapsules containing only *β*-cells and 2.9% in microcapsules, which also included MSCs and RGD (Laporte et al., 2020). This study concluded that RGD incorporation in the alginate microcapsules and co-culturing with MSCs could remarkably improve cell viability and functionality.

#### Fiber-based scaffolds

Fiber-based microdevices are another class of encapsulating devices consisting of non-woven nanofibrous structures in the thickness of micron to macro. However, regardless of device dimension or thickness, cells touch the nanofibrous substrate in nanodimension and cell-substrate interactions are affected by nanosized features in such a device. In recent years, biomimetic nanofibrous scaffolds that are capable of imitating fiber morphology and the biochemical cues of ECM architectures have attracted much more attention in regenerative medicine. Among different approaches for nanofiber formation, electrospinning drew a great deal of interest due to its high throughput and straightforward application (Chen et al., 2018; Al-Enizi et al., 2018; Ojaghi et al., 2019).

In another study, Nassiri et al. have shown that collagen-coated polyethersulfone (PES) nanofibrous scaffold could improve the differentiation of human-induced pluripotent stem cells (IPS) into *β*-cells. In this study, they have used electrospinning to prepare the nanofibers and then modified the nanofibers’ surface with collagen by plasma treatment. Then, IPSs were seeded on the scaffold, and their differentiation, biocompatibility, and insulin release were studied. *In vitro* MTT assay showed 50% cell viability after 7 days of culture. Evaluated results showed a significantly higher gene expression in the seeded cells. The cells that were cultured on the nanofibrous scaffold released significantly higher insulin in medium and high concentrations of glucose than that in the cells cultured on the tissue culture plate (Mansour et al., 2018).

In a recent study, Ruhela et al. have shown the applicability of electrospun nanofibrous substrate for *β*-cell transplantation. In this study, they have used an electrospinning device to prepare polytetrafluoroethylene (PTFE), PES, and cellulose acetate (CA) membrane for *β*-cell encapsulation (Ruhela et al., 2021). They have shown that by increasing hydrophobicity of membrane, cells’ attachment decreased, and *β*-cells aggregate more in this fiber-based ECM-like environment.

### Nanoparticles

**FIGURE 4:**
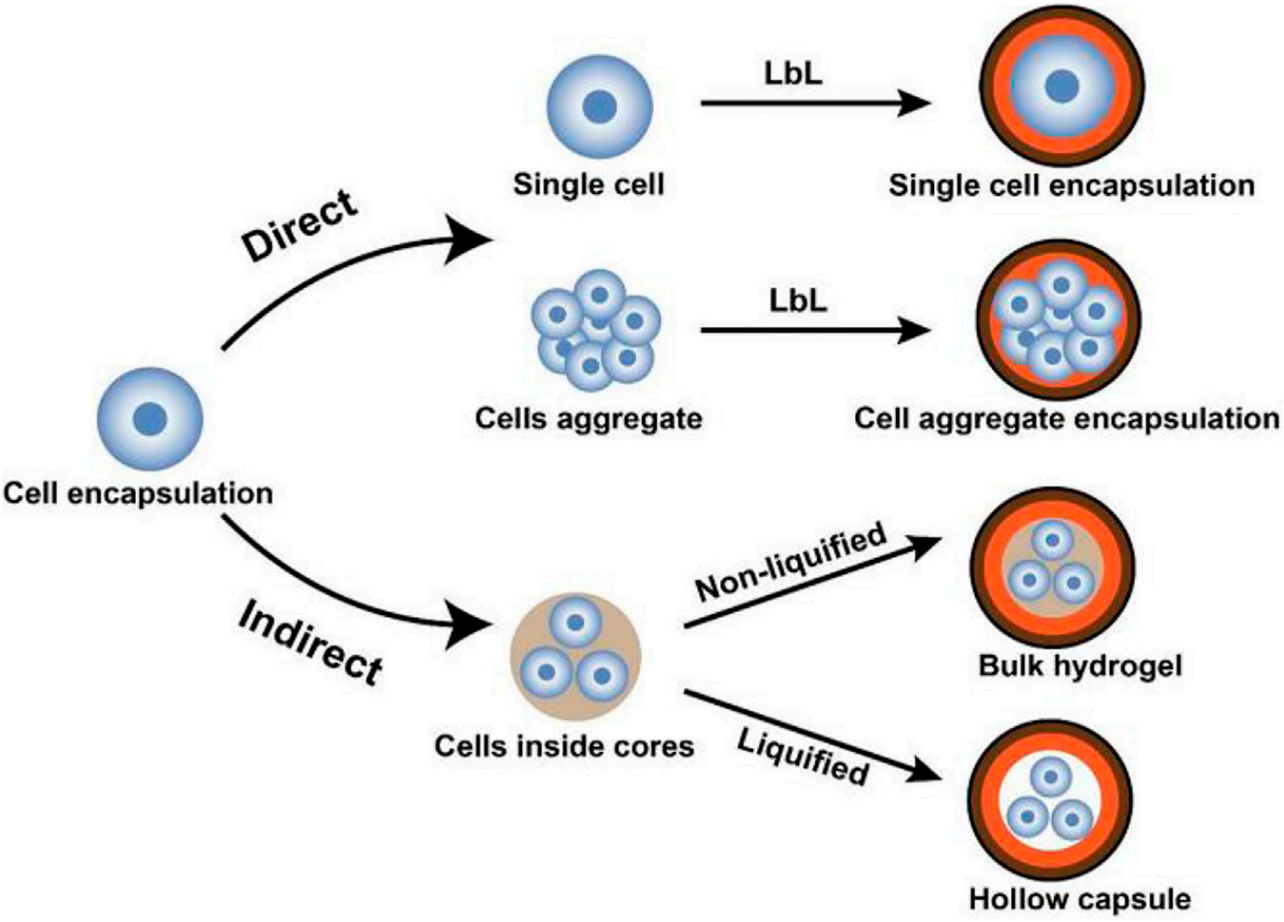
Schematic representation of LBL encapsulation of cells. In the direct approach, cells (single or aggregate) are coated by encapsulating materials, whereas, in the indirect approach, cells are initially encapsulated in core materials then covered by LBL self-assembled shells (Liu et al., 2018).

Encapsulating *β*-cells in nanoparticles is a promising approach by which biocompatible materials coat cells at the nanoscale range. The nanoscale size of these particles enables the facility to inject them into the bloodstream directly. Conformal coating by layer-by-layer (LBL) encapsulation technique is a versatile and tunable technique for encapsulating *β*-cells in the nanoparticles (Dimitrioglou et al., 2019; Krol et al., 2020; Liu et al., 2019). However, the LBL self-assembly technique could be utilized for a single-cell or cell aggregate encapsulation, directly or indirectly, as shown in Figure 4.

Conformal coating of *β*-cells consists of a biocompatible hydrogel covering the cell(s) by forming a crosslinked structure on the cells’ surface. A hydrogel layer covers the cells in the core as a shell. LBL encapsulation technique is based on the self-assembly of polymers as multilayer shells with different charges (polyanion and polycation) on cells’ surfaces (Krol et al., 2020). PEG and alginate biomaterials are widely used for *β*-cell coating.

Recently, single-cell encapsulation has been used for *β*-cells. The major advantage of the single-cell encapsulation technique is its ability to rectify cells’ durability and permselectivity. Durability means increasing cells’ viability by protecting them from dehydration and mechanical stresses. Permselectivity, or selective permeability, means that the artificial, porous coating around cells lets them transfer biomolecules and nutrition selectively (Youn et al., 2020). Techniques used for nanoparticle preparation are similar to those discussed for microparticle preparation. In what follows, the investigation of recent studies, the advantages of single-cell and multi-cell nanoencapsulation techniques in increasing islet cell survival and functionality are discussed.

In a study, Syed et al. have utilized an LBL nanoencapsulation technique to encapsulate *β*-cells in chitosan nanoparticles. They studied islets functionality and viability in *in vitro* and *in vivo*. *In vivo* functionality of islets was assessed by an intraperitoneal glucose tolerance test 15 days after transplantation. The *in vivo* study showed that blood glucose in mouse models treated with nanoencapsulated islets was about 100 mg/dl less than models treated with non-encapsulated cells. *In vitro* assessment of the functionality of nanoencapsulated and non-encapsulated islets exposed to low glucose concentration showed that insulin release from nanoencapsulated cells was about 20 µU/ml more than that from non-encapsulated ones. However, there was no significant difference in insulin release when they were exposed to high glucose concentration. Also, results showed no significant difference in cells’ viability between non-encapsulated cells and nanoencapsulated cells. *In vivo* immunocytochemistry assessment, 4–5 weeks after transplantation, indicated that the morphology of the encapsulated cells appeared to be better preserved than that of non-encapsulated islets (Syed et al., 2018). Therefore, taken together, it could be concluded that nanoencapsulation provided better functionality for transplanted islets.

Encapsulation of stem cell-derived islets in PEG-based nanoparticles was evaluated by Stock et al. to demonstrate the applicability of the encapsulation system in increasing cells’ functionality and their potency to be used as an alternative for islets replacement. *In vitro* assessment of cells’ viability showed that nanoencapsulated cells were as metabolically active as non-encapsulated cells. Although GSIS profile also showed no significant difference in insulin release between encapsulated and non-encapsulated cells, long-term assessment of cells functionality (78–102 days after transplantation) demonstrated that the blood glucose level reported for transplantation of the encapsulated cells was about 58 mg/dl, which was significantly less than that of the non-encapsulated cells (Stock et al., 2020).

### Current Strategies of Engineered Hydrogels for the Improvement of Insulin Secretion

Despite many attempts, the percentage of diabetic patients has been steadily increasing worldwide. Thus, various bio-engineered therapeutic strategies have been established, aiming to achieve more efficient results. It is promising that the encapsulation of islets in hydrogels can control blood glucose level for a long period. However, this goal has not been achieved because of the main hurdles of islet encapsulation, lack of vascularization around islet graft, and activation of host immune response. In order to overcome these obstacles, some strategies are discussed in the following section.

### Improving Biocompatibility Nature of Hydrogels

Biocompatibility is the most vital feature of hydrogel-based biomaterials as it provides appropriate interactions between cells and the scaffold. Regarding islet transplantation, the concept of improving biocompatibility could be discussed in two points of view. From an encapsulated cell/matrix interaction point of view, biomaterials encapsulating the islets could be modified in order to increase cell-biomaterial interactions toward better cell adhesion, cell proliferation, and consequently more cell survival. Also, by itself, biomaterial should not provoke immune reactions and preferably support cell proliferation and their bioactivity (Saroia et al., 2018; Samojlik and Stabler, 2021). The bulk and surface properties of biomaterials, such as topography, wettability, and charge, may be designed to facilitate cell adhesion, proliferation, or differentiation (Amani et al., 2019).

From another point of view, host immune reactions against the transplanted cells are commonly treated by immunosuppressive medications. Improving the immunomodulatory characteristics of the encapsulating biomaterials may result in improvement of biocompatibility and increased survival of the transplanted cells. Immunomodulation may be used to adjust the host immune response if healthy cells become targeted and damaged. On the other hand, considering immune systems reaction against the transplanted cells, which is commonly tailored by immunosuppressive medication after transplantation, resulted in improving biocompatibility and survival rate of cells, while improving immunomodulatory characteristics of the encapsulating biomaterials indirectly. Immunomodulation is the adjustment process of the immune system when healthy cells become targeted and destroyed wrongly via host immune responses (Lebish and Moraski, 1987). Looking at the literature, for islet delivery and encapsulation, the intended biomaterials have been modified to improve biocompatibility and achieve a better result by various strategies. However, regarding the importance of immune rejection concern, much more attention has been paid to improve biocompatibility via immunomodulatory-based designs. In a recent report, Kharbikar et al. have comprehensively reviewed and highlighted the various strategies and techniques that have been utilized for modulating the foreign body response of implants for diabetes (Kharbikar et al., 2021).

In T1DM, as a complex immunological disease, the impaired immune regulation resulted in the autoimmune destruction of *β*-cells (Diana et al., 2011). When islets are transplanted into the patient’s body by biomaterials, the reaction of the immune system against the delivered cells, as well as the delivering biomaterials, serves as a serious barrier and elicits inflammatory reactions. Modulation of immune responses may control inflammatory reactions against the cells or biomaterials and can be utilized to provoke tissue regeneration (Julier et al., 2017).

Hydrogels are popular biomaterials because of their proper biocompatibility and more flexibility in design. These characteristics are originated from their hydrophilic nature and high swelling ratio, leading to an excellent capability of providing oxygen, nutrients, and metabolites and waste products permeability (Singh and Peppas, 2014). Many strategies toward hydrogels can regulate the response of the immune system. Modification of material chemistry incorporation of ECM components or peptides and delivery of anti-inflammatory agents or growth factors as bioactive molecules within hydrogels are generally utilized in this regard.

#### Immunomodulatory Hydrogels

Hydrogels can be modified to modulate immune system responses. In this regard, they have been chemically functionalized with some peptides or ECM components or have been incorporated by some immunomodulatory molecules, which can be released out and affect the immune responses. In some cases, both strategies have been simultaneously utilized in one system. Here, these two approaches are briefly introduced and discussed.

• Immune modulation by delivery of bioactive molecules

Delivery of bioactive anti-inflammatory molecules by incorporation into the biomaterial has been shown to be a promising effective therapy to facilitate tissue productivity, modulate the immune response, and eliminate the need for lifelong immunosuppression. However, controlling the release rate, carrying multiple agents, and supplying a localized and prescribed kinetic of release, seem necessary to achieve adequate immunoprotection by such modified hydrogels (Wang et al., 2019a). Usually, diffusion-based systems, reservoir or matrix, are utilized to sustain the delivery of immunomodulatory factors. In the reservoir delivery system, a drug-containing core is coated by a hydrogel membrane. In contrast, in the matrix devices, drug molecules are dissolved or dispersed in a continuous polymeric matrix, and diffusion is carried out *via* the macromolecular pores or mesh of the matrix (Narayanaswamy and Torchilin, 2019).

Considering the nature of delivered molecules, low molecular weight molecules are released faster from the hydrogel matrix of the membrane, and hydrophilic ones are held more tightly and released at a slower rate (Mayr et al., 2018).

Immune modulatory molecules prevent the destruction of *β*-cells with specific and non-specific actions to restore self-tolerance and alter the immune system’s duration, type, scope, and competency (Lebish, and Moraski, 1987). Such molecules in cell delivery systems can induce much more biocompatibility as they affect the immune responsive cells such as lymphocytes, macrophages, neutrophils, natural killer (NK) cells, and cytotoxic T lymphocytes (CTL). The regulation process by immunomodulatory molecules starts by interfering with the cells’ production of soluble mediators (e.g., cytokines) and could aid cell-cell communication in immune responses by many T regulatory cells, including CD4^+^, CD25^+^, and FoxP3+ (Bascones-Martinez et al., 2014).

Immunomodulatory agents such as anti-rejection drugs are commonly used after the transplantation of islets in order to reduce the rejection probability. However, some of them, including Daclizumab (Zenapax), Sirolimus (Rapamune), and Tacrolimus (Prograf), have noticeable side effects and induce abnormally differentiation (e.g., cancers) (Moini et al., 2015). To prevent the undesired reaction of the immune system against the delivered islets, the selection of appropriate drug combinations is vital for the long-term survival of pancreatic cells.

In various studies, monoclonal antibodies, cytokines, chemokines, and growth factors are incorporated into the hydrogels to modulate the immune responses against the encapsulated islets and enhance the cell viability and biostability. On the other hand, the interaction of such biomolecules with hydrogel matrix, particularly growth factors, also improves their bioactivation and biostability over time. Also, such interactions may foster controlling their sustained release rate from hydrogel substrate (Guziewicz et al., 2011). Nagy et al. have indicated that hydrogel plus heparin could treat autoimmune type 1 diabetes by inducing the sustained release of IL-2 (cytokine), which leads to an increase of T cells proliferation and provoking the expression of forkhead box P3-positive (FOXP3+) Tregs, but soluble IL-2 could not (Nagy et al., 2020). Chemokines like CXCL12 coated on islets or encapsulated with islets in alginate microcapsules resulted in allograft/xenograft islets survival and better function (T. Chen et al., 2015). Other immunosuppressant factors such as TNFα, CD200, TGF-β, CTLA4, and Fas ligands can immobilize onto the surface of the islet and manipulate the function of T cells by deactivating them. For example, when Fas ligand (a type II transmembrane protein) binds to its receptor (CD95), helper T cells die (Desai and Shea, 2017).

**FIGURE 5:**
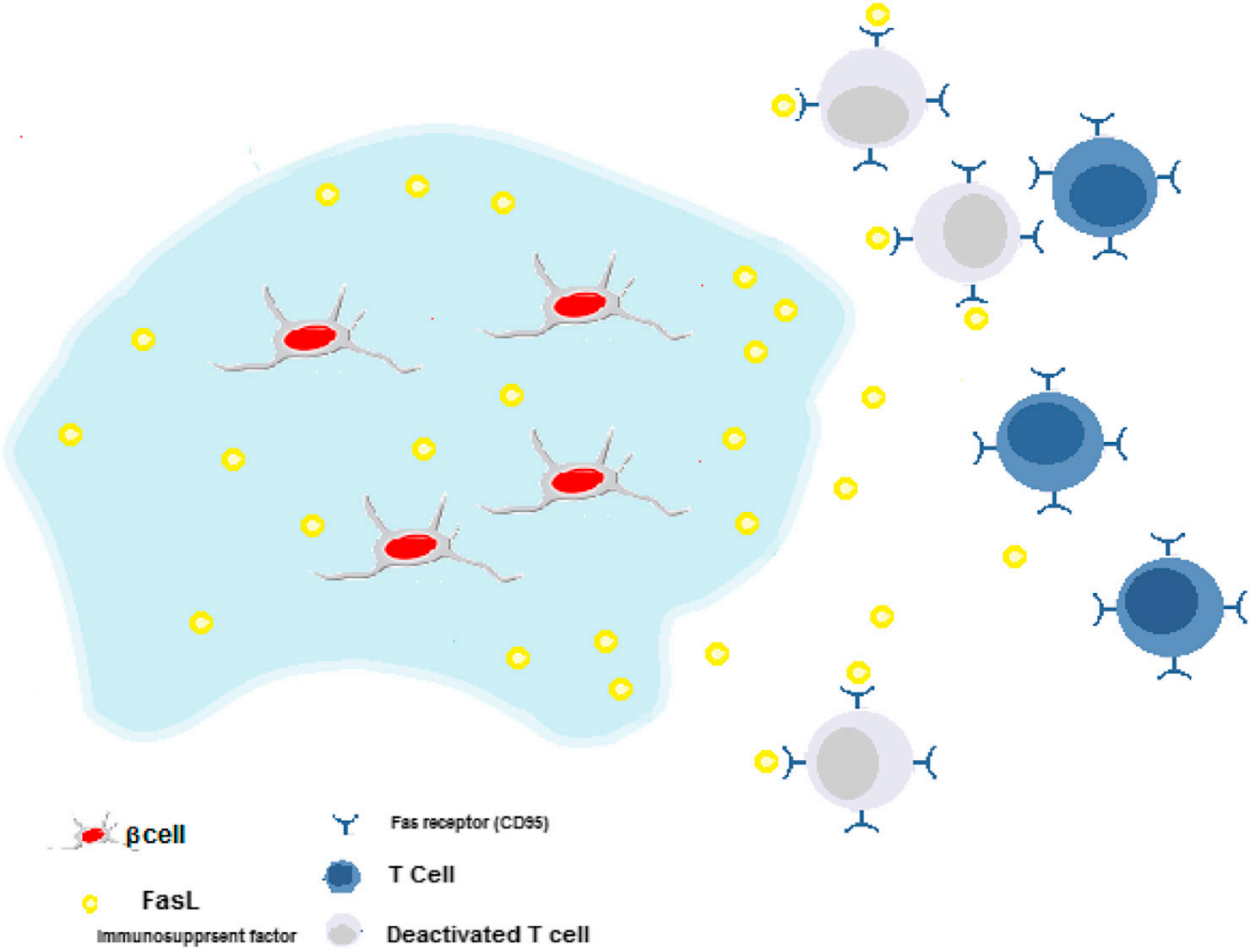
Immune-suppressive hydrogels for delivery of *β*-cells. Cytotoxic T cells around transplantation site deactivated by the release of FasL. This process induces more pancreatic cell survival.

Co-culturing cells that could secrete immunomodulatory biomolecules and islets could also improve these cells' functionality. For instance, Lau et al. have evaluated co-transplantation of FasL-myoblasts with islets. They have shown that the glucose concentration in the blood was normal in a sustained manner and consequently, this process induced more pancreatic cell survival (Lau et al., 1996). It was due to the role of FasL in the protection of islet grafts from immune rejection. Figure 5 indicates the release of FasL from hydrogels and the process of deactivation of cytotoxic T effector cells.

CD200 is a type-1 transmembrane glycoprotein that targets myeloid and lymphoid lineage immune cells (e.g., T cells and macrophages). To prevent all interactions of host immune cells with the transplant, studies have shown that presenting CD200 receptors on the islet coating materials may result in inflammatory suppression and modulation of macrophage actions (Gorczynski 2005; Stabler et al., 2019).

Growth factors also have an important regulatory function in the immune system. Lack or increase in the presence of some growth factors induces diabetes (Shi et al., 2018). Generally, these molecules are produced by many cells and can be restored by ECM components to present the cell surface receptors and have an influence on tissue growth, maturation, and repair (Mitchell et al., 2016). As mentioned before, similar to other immune modulatory factors, a suitable concentration of GFs is necessary for provoking regeneration responses. Regarding *β*-cells, insulin-like growth factor 2 (IGF-2) could promote islet survival and function. For example, Jourdan et al. have co-encapsulated pancreatic islets and bioengineered IGF-II-producing cells in alginate microcapsules to improve islet survival. The results of *in vitro* and *in vivo* studies demonstrated significantly higher cell viability and maintenance of normoglycemia for co-encapsulated samples compared to islet transplanted cells alone (Jourdan et al., 2011).

Fibroblast growth factor 21 (FGF21) is another promising GF that acts as a glucose and lipid metabolism regulator and preserves *β*-cell function (Li et al., 2018; Wang D. et al., 2020). Vascular endothelial growth factors (VEGF), known as the vascular permeability factor, are considered for *β*-cell vascularization (Wirostko et al., 2008). The insulin-like growth factor-I (IGF-I) is a natural polypeptide that has been implicated in diabetic glomerular and renal tubular injuries (Shi et al., 2018). Like other potent regulatory GFs, the TGF-β family affects multiple cell types of the immune system, mediating pro-inflammatory or anti-inflammatory responses. Studies have shown that incorporating this TGF-β in islet encapsulating systems might significantly improve restored normoglycemia and prolonged survival of allogeneic islets. For example, TGFβ1 released from PLGA microparticles encapsulating allogeneic islets enhanced islet engraftment in a chemically induced diabetic mouse model. It is originated from local suppression of pro-inflammatory cytokines (TNF, IL-12, and monocyte chemoattractant protein 1 (MCP1)) (Liu J. M. H. et al., 2016). Hepatocyte growth factor (HGF) is another factor produced by a stromal and mesenchymal cell and provokes epithelial cell proliferation and angiogenesis in many tissues. HGF is a cytokine that enhances motility and morphogenesis (Cao and Wang, 2014). In a study reported by Liu and his colleagues, HGF was delivered by a self-assembling peptide/heparin (SAP/HEP) hybrid hydrogel to prevent inflammatory responses against *β*-cells. The HGF-loaded hydrogel promoted cell viability and reduced *β*-cell death induced by a function of TNF-α (Liu J. et al., 2016).

• Immune modulation by hydrogel chemical modification

The response of the host immune system against hydrogel encapsulating cells often leads to the failure of encapsulated islet graft. One of the typical approaches is transplantation in sites in which immune system reaction is almost muted. Various studies have suggested chemical modification of materials to control host tissue response and avoid fibrosis tissue formation around the transplant site. Alginate-based biomaterials are one of the most prevalent hydrogels suggested to be used for microencapsulation regarding this concern (Goncu and Yucesan, 2020). Zwitterionic polymers are conjugated onto the surface of alginate hydrogel microspheres to reduce the foreign body host tissue reaction by decreasing fibrosis (Q. Liu et al., 2019). Carboxy betaine-based polymers, wherein each monomer was modified by zwitterionic chemistry, caused a low fibrotic response in the subcutaneous site of immune-competent mice (Zhang et al., 2013). It is noteworthy that the chemical modification of hydrogels may affect their other characteristics, such as mechanical properties, and could also impact cell responses indirectly. For instance, Jansen et al. have examined the zwitterionic phosphorylcholine conjugated to PEG hydrogels for islet encapsulation, and the results have shown that the stiffness of hydrogel had a striking influence on the foreign body response, where the stiffer hydrogel elicited fewer responses (Jansen et al., 2018). It has been shown that the immune-protective effect could be achieved by balancing in charge of a hydrogel system when it is essentially uncharged. Hydrogels with neutralized charge may prevent the aggregation of the proteins and macrophages and elicit fewer immune reactions (Zhang et al., 2019). Huang et al. have synthesized an immune-shielding thread-like hydrogel based on the alginate-polyethyleneimine (PEI) for islet encapsulation. Melanin nanoparticles as a NIR-responsive material were incorporated into the hydrogel in order to stimulate the secretion of islets with or without a NIR treatment. Results have shown excellent immune-shielding properties against the encapsulated islets *via* utilizing charge neutralization of alginate by PEI and melanin. In addition, incorporated melanin stimulated insulin secretion, particularly under exposure to NIR, and the islets showed functional physiological activity. However, the mechanism of how melanin can improve insulin secretion still needs more investigation (Huang et al., 2021).

Recently, Hu et al. have designed alginate-based bioinks (alginate, alginate supplemented with Pluronic 127, and alginate combined with pectin) for *β-*cell encapsulation with immunomodulating capacity. An extrusion-based 3D printer was used for layer-by-layer bioprinting of these bioinks. The influence of the pectin incorporation on beta islet cells viability under inflammatory stress was assessed by exposing the cell-containing hydrogel to a cocktail of the mouse cytokines, IFN-γ, TNF-α, and IL-1β.Live/dead staining was also used for studying the beta islet cell viability. Results of the viability study showed an almost 9% increase in cell survival for pectin-incorporated bioink. *In vivo* tissue response also was assessed by analyzing fibrotic overgrowth thickness. The layer of fibroblast found on the pectin-incorporated construct was about 89 µm, while for alginate-Pluronic, it was about 172 µm. This result indicates the significantly higher biocompatibility for the pectin-incorporated hydrogel (Hu et al., 2021).

• Immune modulation by hydrogel mechanical and topographical modification

Mechanical stability is a substantial need for long-period transplantation of hydrogel-based encapsulated cells. The insufficient mechanical properties of the capsules may result in instability of them in physiological condition; moreover, pieces of evidence demonstrate that immunological responses are provoked in the recipient *via* mechanical instability. For instance, as mentioned, increasing stiffness of PEG-based encapsulating hydrogel decreases foreign body reactions (Jansen et al., 2018).

Since most of the hydrogels are weak regarding the mechanical properties, their mechanical features could be modulated by adding covalent crosslinking to the structure, grafting, or blending with other suitable polymeric chains in order to prevent the destruction of the capsules and long-term biocompatibility. On the other hand, as a biophysical feature of the environment, most of the cells can sense the mechanical nature of the surrounding environment and behave correspondingly (Huebsch, 2019). Therefore, tuning the mechanical properties of hydrogel could serve as a strategy to modulate encapsulated cell behaviors. Richardson et al. have evaluated the effect of stiffness of barium-alginate capsule on growth, the viability of human embryonic stem cells (hESCs), and their differentiation to pancreatic islets (Richardson et al., 2016). They have prepared alginate capsules with different stiffness via cross-linking in various concentrations of BaCl_2_ solution (10–100 mM) as a cross-linker. Results interestingly demonstrated that hESCs growth and differentiation were strongly dependent on the stiffness of the hydrogel where the stiffness of approximately 4–7 KPa was most supportive of cell proliferation, while stiffness around 3.9 KPa was best for pancreatic differentiation. However, much increases in the stiffness strongly suppressed pancreatic progenitor induction. Although a few studies have focused on investigating mechanical characteristics of islet encapsulating hydrogels, their great importance in cells-substrate interactions need to be considered.

Beyond the material’s chemical and mechanical properties, structural and topographical features in micro- and/or nanoscale (e.g., size, shape, and geometric alignment) may play a crucial role in directing cell behavior such as adhesion, proliferation, migration, arrangement, and differentiation (S. Wang et al., 2019b). In addition, proper microstructural features or topographies can modulate immune system function. For instance, Tylek et al. have studied the effect of pore size in fibrous scaffolds on macrophage polarization and subsequently its phenotype, pro-inflammatory (M1) or pro-healing (M2) (Tylek et al., 2020). They have fabricated a series of 3D porous fibrous scaffolds with various pore sizes from 40 to 100 µm by melt electro-writing, followed by seeding of primary human peripheral blood-derived macrophages. Interestingly, their observations demonstrated that pore size affected macrophage elongation and transition (M1 to M2 phenotype) where the smallest pore size of 40 μm was beneficial for enhancing both the elongation and polarization of human macrophages toward the pro-healing phenotype rather than pro-inflammatory one. The microscale dimensions may play an important role in the modulation of immune responses; moreover, evidence also showed that in macroscale, foreign body reactions are affected by the size of implanted biomaterials. Veiseh et al. have shown that alginate spheres encapsulating islet cells with a diameter in the range of 1.5–2.5 mm had a significantly decreased foreign body response compared with smaller diameter spheres (<1 mm). In addition, their *in vivo* study demonstrated that rat pancreatic islets are encapsulated in 1.5 mm alginate capsules and are transplanted into streptozotocin-treated diabetic C57BL/6 mice, which restored blood glucose control for up to 180 days, whereas for transplanted capsules in the size of 0.5 mm, blood glucose regulation was restored for a significantly shorter period (∼35 days) (Veiseh et al., 2015).

**FIGURE 6:**
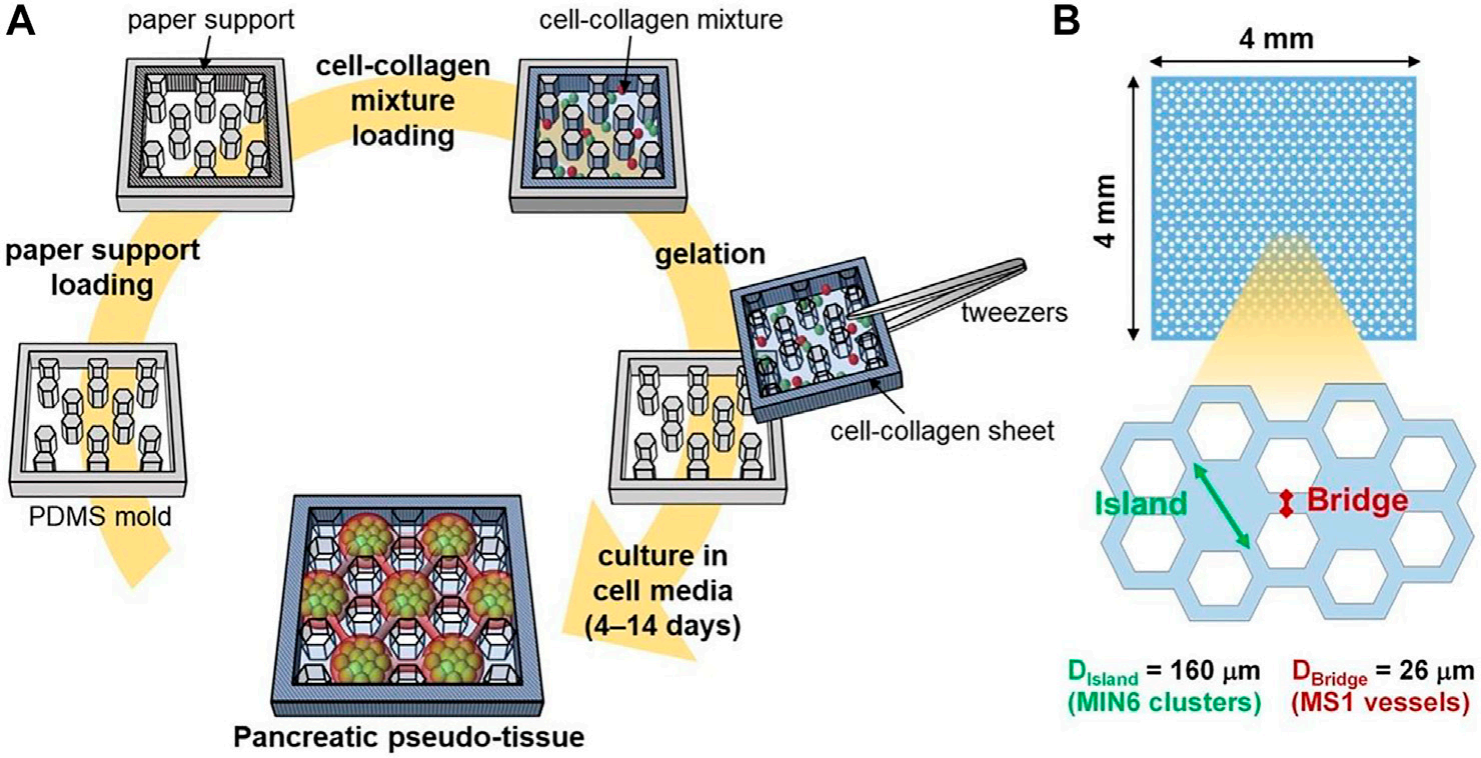
**(A)** The procedure of preparation of micro-patterned collagen sheet as a pancreatic pseudo-tissue and **(B)** dimensions of hexagonal micro-pattern (Seo et al., 2020).

**FIGURE 7:**
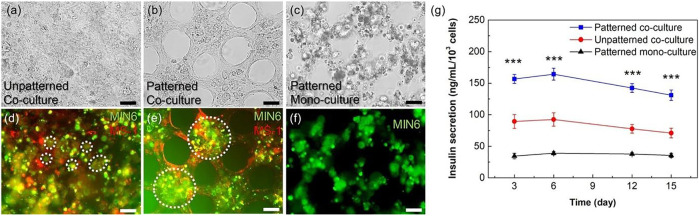
Evaluation of the micro-patterns effect on cellular organization and function. Microscopic images of MIN6 and MS1 co-cultured sheets without **(A, D)** and with pattern **(B, E)**. Microscopic images of a MIN6 mono-cultured sheet with micro-patterns **(C, F)**. In fluorescence images, MIN6 cells are stained in green, and MS1 cells are stained in red. Cellular clusters are pointed by white dashed circles. **(G)** Insulin secretion level of MIN6 cells in mono- and co-cultured condition (patterned and non-patterned). Scale bars are 50 μm, and *p*-value <0.001 indicates significant differences (Seo et al., 2020).

Regarding the importance of topographical feathers in cellular responses, this strategy may be utilized in order to modulate pancreatic cell function. Seo et al. have designed a micro-patterned collagen sheet in order to mimic a microstructural model of pancreatic tissue (Figure 6) (Seo et al., 2020). They have co-cultured mouse pancreatic *β*-cell (MIN6) and mouse pancreatic islet endothelial cell (MS1) within the micro-patterned sheet and evaluated the cell organization and function compared to cells cultured in a non-patterned substrate. Results have shown that in the patterned culture model, during 4–10 days, the MIN6 cells formed islet-like clusters are surrounded by an endothelial MS1 cell monolayer (Figure 7). In addition, interestingly, the MS1 cells made a connection between the clusters, resembling a blood vessel-like structure. In contrast, the 3D co-culture structure was not formed in a non-patterned collagen sheet. The organization of cells was affected by micro-patterning, and insulin secretion by islet-like clusters was also improved, where the insulin secretion level of MIN6 cell-cultured within micro-patterned sheets was significantly about 1.5 times higher than that of those cultured within non-patterned sheets.

#### Bioactive Hydrogels

In the pancreas, each islet is surrounded by a native ECM. During cell isolation, most ECM components are destroyed, and cell viability and function are affected after transplantation in the new site. (Stendahl et al., 2009). Transplantation of the isolated or cultured cells via a suitable substrate that keeps cells interactions with the microenvironment is a better approach than transplantation via an inert substrate. Hydrogel functionalized with biomolecules including protein or peptides can promote cell adhesion and survival in cell transplantation. Utilizing these adhesive molecules promoted the adhesion receptors (integrins)-cell interactions (Hynes 2002). Collagen IV is the main structural component that maintains *β*-cell attachment, migration, proliferation, differentiation, and also survival (Yap et al., 2013). Fibronectin induces pancreatic cells’ viability and function by preventing apoptosis and laminin, resulting in growth in glucose-stimulated insulin secretion. Incorporating such ECM-derived components into hydrogels designed for islet cell encapsulation significantly increases the effectiveness of such treatments (Llacua et al., 2018).

For example, although PEG-based hydrogel has been successfully studied in *in vitro* islet encapsulation and transplantation, it cannot survive *β*-cells for a long time, mainly because of the lack of cell adhesion ligands and interactions of cells with the surrounding microenvironment (Ouyang et al., 2019). In a study, Weber et al. have assessed the role of incorporation of ECM-derived proteins (collagen type I, collagen type IV, fibrinogen, fibronectin, laminin, and vitronectin) into the 3D PEG environment on islets survival. The overall result demonstrated that incorporating these crosslinked proteins decreased cell apoptosis significantly after transplantation (Weber et al., 2008).

For example, modification of enzymatically crosslinked protein-based hydrogels by incorporation of ECM-derived proteins (collagen IV, fibronectin, and laminin) for encapsulation of MIN6 *β*-cells was investigated by Liese N. Beenken-Rothkopf et al. The results demonstrated that involvement of ECM proteins in the hydrogel matrix maintained high insulin response compared to cells encapsulated in the no-modified hydrogel at day 7. However, the concentration of modifier EMC proteins was also an important issue that needed to be optimized (Beenken-Rothkopf et al., 2013).

Besides proteins, peptides are a significant modifier that can induce cell signaling when interacting with the corresponding receptors. Peptides also show more benefits than proteins due to their low cost, the simple synthesis procedure, and more resistance to many environmental conditions. Peptide-modified structures present structural and biological traits with high similarity to protein-modified substrates for cell encapsulation applications. Many peptides can carry out the function of ECM proteins and some GFs, as well. However, for some proteins, protein-derived peptides cannot perform as efficiently as the protein (Klimek and Ginalska, 2020). For example, Weber and Anseth have shown that the addition of whole ECM proteins enriched by laminin in PEG dimethacrylate (PEGDM) hydrogel encapsulating *β*-cells results in the secretion of more insulin compared to PEGDM hydrogel modified with laminin-derived peptides (Weber et al., 2008) because of the limited effect of short peptide moieties compared to the whole ECM proteins.

Nevertheless, the inclusion of these molecules into the polymeric biomaterials mainly results in their enrichment with pro-adhesive sequences. Many proteins and peptides have pro-adhesive sequences such as RGD, PHSRN, YIGSR, and IKVAV. RGD is one of the most widely considered adhesive peptides, which is effectively modified to enhance cell attachment to many surfaces and substrates. RGD immobilization directly onto biomaterials enhances the adhesion of receptor-mediated cells to the surface of biomaterials (Klimek and Ginalska, 2020). However, it is noteworthy that immobilized peptide density and orientation are key parameters affecting cell attachment (Bellis, 2012). A study reported by Joana Crisostomo et al. RGD with a concentration of 200 μM was incorporated into alginate hydrogels (2% m/v) and provided a profitable niche for *β*-cell survival and consequently insulin secretion (Crisóstomo et al., 2019). In another study, regarding the potential of semi-permeable PEG as an immune-isolation barrier encapsulating islets, glucagon-like peptide-1 (GLP1) was immobilized within PEG hydrogels and increased *β*-cell durability, survival, and insulin secretion compared to unmodified-PEG. GLP-1 is already known to provoke the expansion of insulin-secreting *β*-cell (Lin and Anseth, 2009). Similarly, a survey on PEG hydrogel, which was functionalized with RGD, IKVAV, and GLP-1, demonstrated the survival of bone marrow MSCs and pancreatic islets by GLP-1 stimulation and increase of glucose level (Bal et al., 2017). Another example of peptide-functionalized hydrogel, IL-1RIP inhibitory peptide-modified hydrogel, increased the survival of encapsulated MIN6 cells by reducing the cells’ apoptosis up to 60%. Jing Su et al. have demonstrated that PEG-based hydrogels (4-armed PEG cysteine and 4-armed PEG thioester) containing this inhibitory peptide could resist the cytokines such as IL-1b, TNF-alpha, and INF-g. Co-presence of IL-1RIP with other adhesion peptide sequences such as GRGDSPG improved the anti-cytokine effects compared to hydrogels containing either of the two peptides alone (Su et al., 2010).

### Improved Vascularization Potential of Hydrogels

As mentioned, islets are highly vascularized tissues in which vascularization is crucial for islets’ function in blood glucose responsibility and insulin secretion. It is noteworthy that blood vessels are lined with a very high density of endothelial cells in this tissue. The formation of this featured vascular system requires that the participation of VEGF-A binds to VEGF receptor (VEGFR)-1 and VEGFR-2, which are also known as FMS-like tyrosine kinase 1 (Flt1) and kinase insert domain protein receptor (Kdr), respectively. These two receptors are critical regulators of vasculogenesis, angiogenesis, vascular permeability, and endothelial fenestration formation (Brissova et al., 2006; Lugano et al., 2017). Through embryonic development, endothelial-endocrine cell signaling and blood vessels’ reconstruction induce pancreatic morphogenesis (Brissova and Powers, 2008).

After isolation and transplantation of islets, blood flow reestablishment for the grafted islets needs time. The transplanted islets would be subjected to hypoxia condition and the lack of nutrients compared to the native pancreas, it undergoes to be necrosis. Indeed, the long-term survival of islets after transplantation needs vascular support. Thus, improving transplanted islets’ revascularization will significantly promote cell survival and secretion of insulin (Pepper et al., 2013). Localized delivery of angiogenic molecules and modulating immune responses at the implantation site by co-transplantation of supportive cells can induce vascular formation (Desai and Shea, 2017).

#### Angiogenesis Aided by Co-Transplanting of Cells

Apart from the microenvironment, cell-cell interaction modulates cell signaling. Co-culture of suitable cells along with islets could support improved cell functions. In this regard, co-encapsulation of angiogenic cells or regulatory cells has been suggested in order to improve islet cell survival and function via supporting angiogenesis. Regulatory T cells (Tregs) are helpful mediators’ immune homeostasis, which can suppress the immune response, and they have a dynamic role in angiogenesis. T cells are identified mainly by those that express CD4, CD25, and FOXP3. It is a well-known approach to transplant *β*-islets with Tregs, mainly Tregs anchored stably to the cell surface (Kryzstyniak et al., 2014). Multipotent adult progenitor cells are another proper candidate for co-transplantation with islets because of their ability to suppress immune cells and promote angiogenic responses (João Paulo et al., 2016). Islets co-transplanted with multipotent adult progenitor cells could induce endothelial and advance neovascularization around islet transplant. Recently, adult MSCs are widely co-transplanted with islets. They promote *in situ* angiogenesis by secreting immunosuppressive molecules such as prostaglandin E2 preventing hypoxia, and suppressing immune cells, including NK cells, macrophages, neutrophils, and T cells (De Miguel et al., 2012).

Bone marrow and adipose tissue are two primary sources of MSCs isolation. These cells have the noticeable potential to differentiate into the various lineages (Mohamed-Ahmed et al., 2018). The co-transplanting of MSCs with the islets has shown highly acceptable outcomes in islets viability and growth of insulin levels after transplantation. For example, Rackham et al. have investigated the effect of islets co-transplantation with MSCs in C57Bl/6 mice on glycemic levels of insulin secretion. Results have demonstrated that 92% of animals got normoglycemia compared to those transplanted with islets alone (42%). Vascular engraftment was also noticeable in MSC co-transplanted mice, as indicated by enhanced endothelial cell numbers within the endocrine tissue. They have shown a significant reduction of glycemic levels and remarkable improvement of blood insulin level *via* co-encapsulation (Rackham et al., 2011). In a similar study, Kerby et al. have assessed co-encapsulation of mouse islets and kidney MSCs in alginate to improve graft outcome in a microencapsulated/isolated graft model of islet transplantation. Results have demonstrated that the average blood glucose concentration significantly decreased by 3 weeks in the co-encapsulated samples. Furthermore, after 6 weeks, compared with the islet-alone group, the co-encapsulated group showed around 4.5-fold increase in curing. Consequently, the efficiency of microencapsulated islets is raised by MSCs co-culturing due to their effect on revascularization and maintenance of islets morphology (Kerby et al., 2013).

In healthy pancreatic islets, endothelial cells constrict blood vessels inside and outside the islets. They are losing these connections between *β*-cells and endothelial cells after isolation, which may interrupt islet functions. Thus, endothelial cells are another candidate for co-transplanting with islets owing to their essential role in maintaining proper signaling between *β*-cells and preserving their viability and function (Hogan and Hull 2017; Skrzypek et al., 2018). The lack of factors produced by endothelial cells likely results in low productivity of *β*-cells and insufficient insulin release (Skrzypek et al., 2018). Sebara and Vernette have indicated that the insulin release of rat insulinoma cells (INS-1) increased when they were co-cultured with human umbilical vein endothelial cells compared to INS-1 cells alone (Sabra and Vermette, 2013).

#### Angiogenesis Aided by Angiogenic Molecules and GFs

Utilizing GFs and angiogenic molecules embedded in hydrogels can provoke angiogenesis and blood vessel development (Nour et al., 2020; Malektaj et al., 2021). Basic fibroblast growth factor (BFGF) is identified to enhance angiogenesis after transplantation into recipients through increasing new capillaries around islet graft (Kawakami et al., 2001; Zhu et al., 2019). Studies have demonstrated the combination of BFGF with collagen hydrogel assist’s revascularization responses and indicated a reduction in islet damage by hypoxia (Kawakami et al., 2001).

Nina Yin et al. have synthesized a VEGF incorporated (100 ng/ml) alginate hydrogel to encapsulate the islets. Results have indicated a sustained angiogenesis promotion in the diabetic mice model. Consequently, islet survival and function improved and exhibited a therapeutic effect over 50 days (Yin et al., 2016a). In a similar study, VEGF stimulated angiogenesis through PEG maleimide (PEG-MAL) hydrogels. It also enhanced graft vascularization, cell viability, and insulin secretion in encapsulated rat islets during 4 weeks (Phelps et al., 2015; Phelps et al., 2013). Connective tissue growth factor (CTGF) is generally expressed in endothelial cells, and downexpression of this GF may cause less vascularized islets. Thus, CTGF can also be utilized as a vasculogenic stimulator (Crawford et al., 2009).

In another approach, incorporating O_2_ producing molecules such as calcium peroxide within a transplanted construct is beneficial. However, it may have some drawbacks, like suppressing the release of angiogenic GFs and a delay in implant vasculogenesis (Camci-Unal et al., 2013). To overcome this issue, such oxygen-producing reagent could be co-delivered with some angiogenesis stimulators.

**FIGURE 8:**
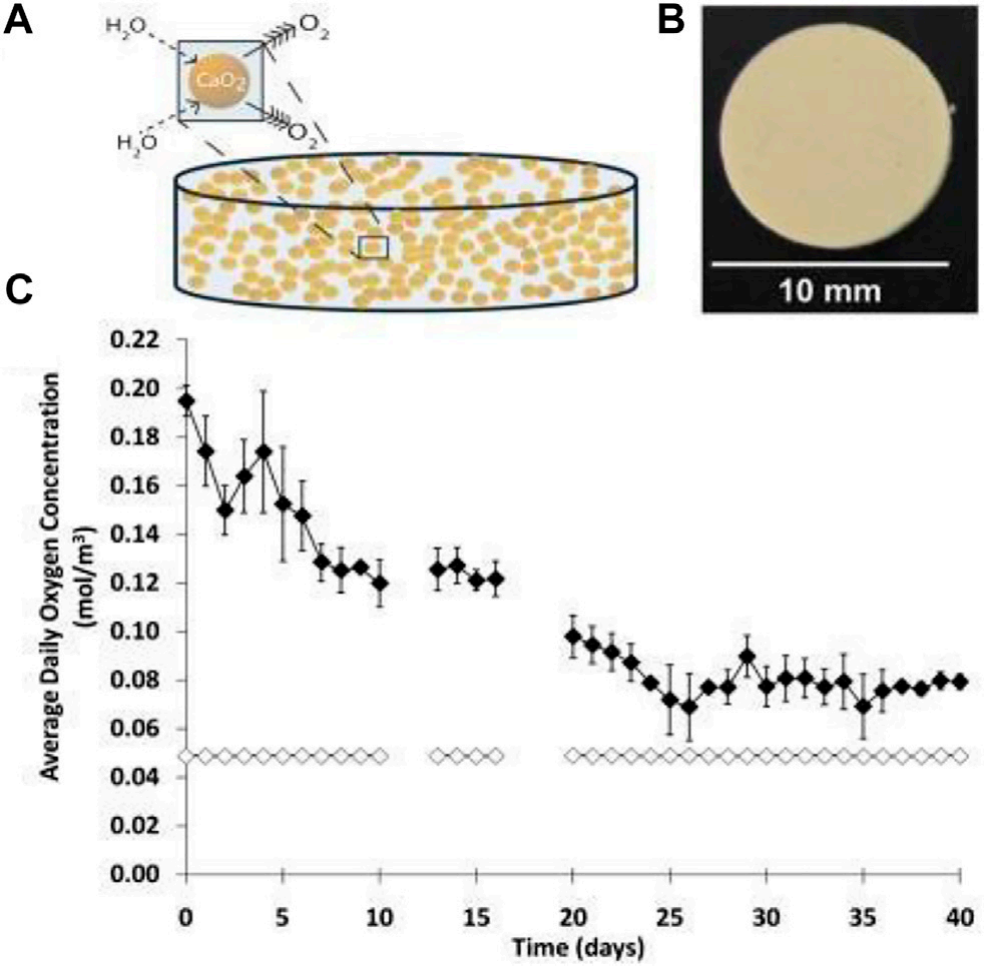
Schematic showing oxygen-generating PDMS-CaO_2_ disc **(A)**. Photograph of PDMS-CaO_2_ disk (10 mm diameter; 1 mm height) **(B)**. Oxygen releasing screening of PDMS- CaO_2_ disk (filled diamonds) compared to blank PDMS disk (open diamonds) during 40 days **(C)** (Pedraza et al., 2012).

In a study reported by Pedraza et al., in order to prevent hypoxia-induced cell death in *β*-cells, they have incorporated CaO_2_ into polydimethylsiloxane (PDMS) discs (Figure 8), which were placed in the core of *β*-cell-laden agarose hydrogel. This combined system provided a sustained release of oxygen for more than 40 days, which supported encapsulated MIN6 *β*-cells and pancreatic rat islets. PDMS-CaO_2_ disk induced glucose-dependent insulin secretion and sustained increase of *β*-cell proliferation for more than 3 weeks in hypoxic culture aside from the fading role of vasculogenesis GFs (Pedraza et al., 2012).

In another study, Wang et al. have reported an inverse breathing system with the ability of oxygen generation from carbon dioxide (CO_2_). The reaction of CO_2_-lithium peroxide from an aqueous cellular environment by a gas-permeable membrane yielded a device that supported functional islets in retrieved mice for more than 2 months (L.H. Wang et al., 2021).

In a recent study reported by Toftdal et al., an oxygen releasing system based on thiolated hyaluronic acid (tHA) and 8-arm PEG-Acrylate (PEGA) was synthesized (Toftdal et al., 2021). This injectable hydrogel was incorporated by calcium peroxide in various concentrations (0, 7.5, and 30%) and evaluated in terms of chemical and physical properties and suitability for *β*-cell encapsulation. Results have demonstrated that the incorporation of calcium peroxide did not interfere with the injectability of hydrogels. However, it decreased the gelation time. Monitoring the oxygen release kinetics under hypoxic conditions showed that hydrogels containing 30% calcium peroxide released oxygen for at least 30 h, whereas hydrogels containing 7.5% calcium peroxide could not support oxygen releasing after 10 h of incubation. In order to assess the effect of oxygen release on cell viability, clusters of insulin-secreting reporter cells (INS-1E) were encapsulated within the designed hydrogels and a live/dead staining assay was conducted on days 1 and 3. The calcium peroxide-laden hydrogels supported the viability of encapsulated clusters over 3 days, while necrosis signs were observed in clusters encapsulated in hydrogels without calcium peroxide.

Providing oxygen supply for a longer time could guarantee more efficacy and success of islet transplantation, particularly for clinical applications. In this regard, Liang et al. have developed a distinct implantable platform containing oxygen-generating micro-beads (Liang et al., 2021). They have used biostable PDMS encapsulating calcium peroxide, termed “OxySite,” in a macro-porous PDMS scaffold as an oxygen-generating device. Data that showed this device generated sufficient local oxygenation for up to 20 days and supported clinically relevant cell dosages (10,000 islet equivalents per kilogram body weight (IEQs/kg BW)) for islet transplantation in a diabetic Lewis rat syngeneic transplantation model. *In vitro* and *in vivo* outcomes demonstrated that the OxySite scaffold had great potential to support increased oxygen demands without delay in vascularization.

Beyond insufficient oxygen and nutrition transport, oxidative stress is also one of the important factors contributing to beta islet cells failure. Oxidative stress is defined as the imbalance between the production of reactive oxygen species and antioxidant defenses in the cells. L. Reys et al. have, in a recent study, addressed this issue for beta islet cells encapsulation in alginate-based microcapsules (Reys et al., 2021). They have investigated the effect of two different algae species, *Fucus vesiculosus* (FF) and *Ascophyllum nodosum* (FA), on alleviating oxidative stress *in vitro*. FF and FA are a kind of fucoidan, which are polysaccharides with the potency of oxidative defense attributed to their sulfate group. In this study, the fucoidan-alginate hydrogel was prepared by mixing the two components and crosslinking them with barium chloride. The antioxidant potential of the final hydrogel was assessed using the total antioxidant capacity assay kit and expressed as Trolox equivalent antioxidant capacity (TEAC), with Trolox serving as an antioxidant standard. Final results indicated that the antioxidant capacity of the FF-alginate microcapsules was 35-fold higher than that of alginate hydrogel. Cell viability and insulin secretion studies have also demonstrated the higher capacity of the FF-alginate hydrogel in *β*cells’ survival and functionality. The results demonstrated 1.8-fold higher insulin secretion and 8.6% more cell viability in FF-alginate hydrogel.

## Clinical Trials of Islet Encapsulation and Commercialized Products

Apart from numerous reports regarding the application of islet transplantation in diabetes treatment, the number of clinical trials is limited, likely due to donor shortage and the need for lifelong immunosuppression (Desai and Shea 2017). These limitations attract attention toward investigation on the clinical applicability of the encapsulated devices.

Whereas micro-/nanoencapsulation devices can transfer the maximum amount of islets, support nutrients, and facilitate insulin secretion due to their thickness and porosity limitations, precise implantation still serves as considerable drawbacks. Some advanced micro-manufacturing technologies, including micro-electromechanical systems (MEMS) such as microfluidics and micro-molding devices, are adopted to prepare micro-/nanodevices with the precise structure for islet encapsulation (Espona-Noguera et al., 2019).

In a clinical study, Soon-Shiong et al. have successfully encapsulated 20000 allogeneic islets in alginate microcapsules and transplanted them into the peritoneum of a patient under immunosuppression. In this study, they have used alginate as a biocompatible immunoprotective membrane to prevent transplantation rejection. The results showed control in glucose metabolism restoration with insulin independence for 9 months (Soon-Shiong et al., 1994). Similarly, in 2006, for two patients, a total of 400,000 (for patient #1) and 600,000 (for patient #2) human islets were microencapsulated in alginate-PLO (poly-l-ornithine) hydrogel transplanted in two diabetic patients. Results have shown a reduction in insulin requirements with an increase in serum C-peptide levels in patients without any immunosuppressants (Ontanucci and Ancuso, 2006). Tuch et al. have also clinically examined the applicability of microencapsulated human islets loaded in barium-alginate hydrogel for four patients treated by any immunosuppressive drugs. The findings of this study showed that blood glucose levels and insulin requirements were significantly lowered after 1 day (Tuch et al., 2009). In 2007, Eliott et al. reported the transplantation of neonatal porcine cells, as promising sources for insulin-producing islets (Dufrane and Gianello, 2012), in the peritoneal cavity of a patient. In this study, 15,000 IEQs/kg body weight were the implant dose and reported results indicated an improvement in HbA1c levels up to 14 months and detectable C-peptide up to 11 months after transplantation. Interestingly, the transplanted capsules included viable islets even 9.5 years after transplantation. In conclusion, using the encapsulation technique demonstrated long-term survival of functional xenogeneic cells transplanted without immunosuppressive medication (Elliott et al., 2007). At the same time, LCT Company transplanted pig islets loaded in alginate-PLO microcapsules in eight patients and observed a considerable decrease in insulin demand, even 8 months after transplantation (Tan, 2010).

**FIGURE 9:**
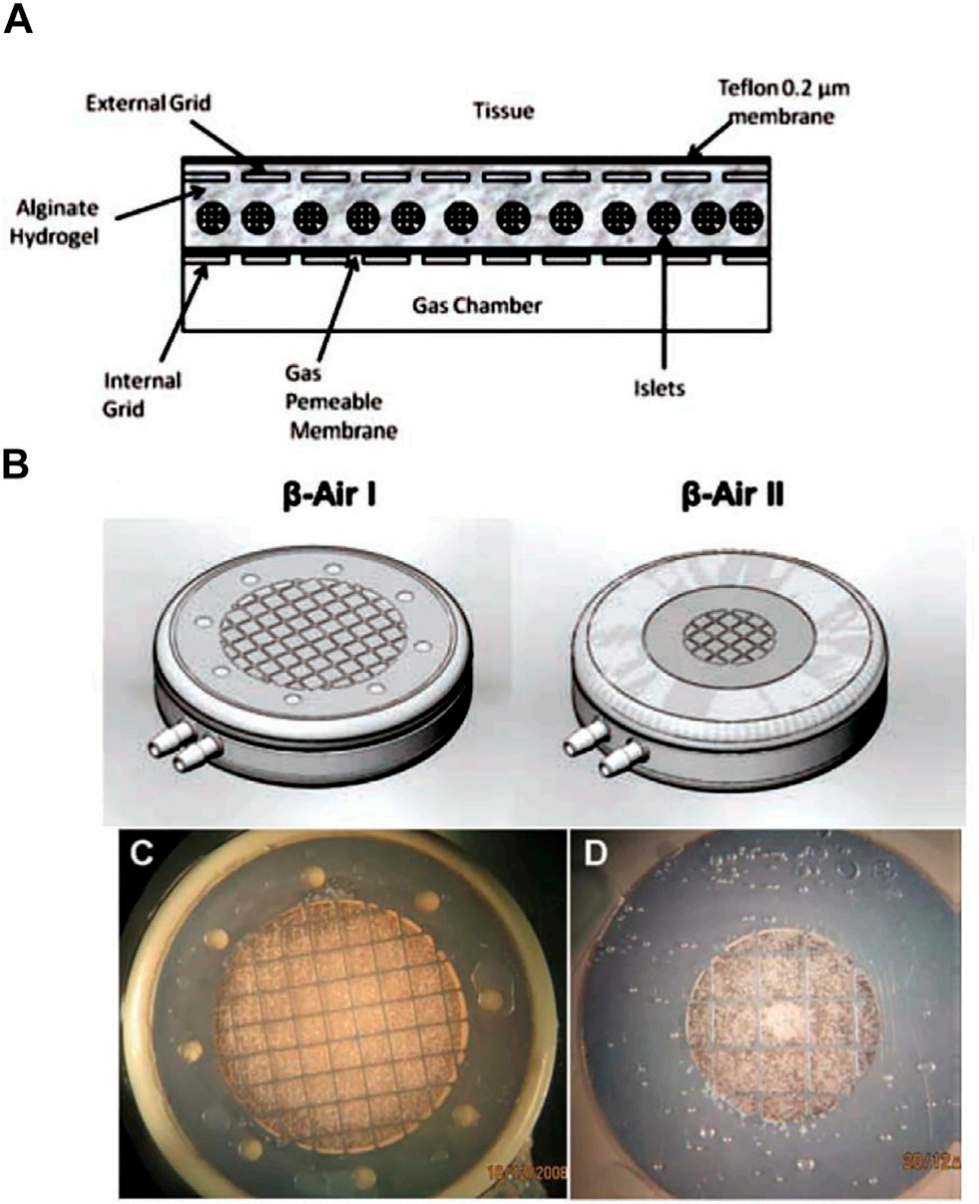
Schematic view of the βAir I and II implantable bioartificial pancreas devices: **(A)** cross-section and **(B)** external view. The β-Air I **(C)** and β-Air II **(D)** modules seeded by islets before implantation (Barkai et al., 2013).

As previously discussed, macroencapsulation devices have some limitations due to their complicated designs, low mass transport, and limited cell response (Espona-Noguera et al., 2019). Furthermore, the slow diffusion of oxygen into this device may cause delays in insulin release leading to hypoglycemia (Izeia et al., 2020). However, macrodevices are safe; they can reduce the risk of teratoma formation, cells’ survival, and their secreted insulin for months. Among limited clinical studies, the bioartificial pancreas βAir device, developed by Beta-O_2_ Technologies Ltd., was studied for macroencapsulation of human islets (Figure 9). In this device, islet-laden material (e.g., alginate, Teflon) encapsulates 10,000 IEQs/kg dosage of islets; besides, the device is coupled with a gaseous oxygen source, which is transplanted under the skin of non-immunosuppressed patients with T1DM. This system allows a controlled oxygen supply to the islet grafts to utilize an integrated oxygen reservoir. This reservoir can be refilled regularly and can maintain oxygen pressure. Results have demonstrated that βAir macroencapsulation devices overcame the challenge of oxygen delivery and maintained human islets survival more than 6 months post-implantation (Ludwig et al., 2013).

Viacyte and many other organizations have reported the successful applicability of TheraCyteTM devices for *β*-cells’ transplantation in patients who did not receive immunosuppressive drugs. TheraCyte device is a planar structure in which islets are seeded between two porous polytetrafluoroethylene (PTFE) immunoisolation membranes in order to stimulate vascularization. This study demonstrated the successful protection of *β*-cells by this device in non-immunized patients, but its biocompatibility has not been studied yet (Kumagai-Braesch et al., 2013). ViaCyte offered two products, the “PEC-Direct” and the “PEC-Encap” devices, for the treatment of patients with severe T1D. The first one had been on the pre-clinical phase (2017), and FDA had approved the second one in 2014 for phase I and II clinical trials. The human pancreatic cells (PEC-01) were encapsulated in these devices and implanted under a patient’s skin in order to create proprietary pancreatic progenitor cells, which are becoming islets. These two products offer a potential functional cure for diabetic patients (Cooper-Jones and Ford 2016). In 2017, Biadal et al. have, from the University of Miami, implanted a total of 602,395 allogeneic islets by fibrin-based biodegradable scaffold onto the omentum of a person who had diabetes type 1 for 25 years. Recipients remained insulin-independent for more than 1 year (Baidal et al., 2017).

BioHub^™^ macroencapsulation devices are also accepted for clinical trials. In a study, allogeneic islets were encapsulated in the BioHub^™^ system and implanted in diabetic women’s body. Using this macrodevice resulted in insulin-independency for 1 year (Kepsutlu et al., 2014).

Although many clinical studies have been conducted for the transplantation of *β*-cells, the exact result of a fully functioning encapsulation approach has not been provided yet. Among macro-, micro-, and nanoencapsulation devices applied for clinical trials, bio-printed devices offer great potential in excluding the undesirable properties of these systems. Using bioprinting technology, scientists can design artificial pancreatic tissues with vascular structures. However, this emerging technology is still in its initial stages and requires further researches (Juewan Kim et al., 2019b).

## Conclusion

Diabetes is one of the most prevalent diseases in the world. Although islet cells transplantation serves as a therapeutic approach in treating T1DM, some challenges need to be overcome to achieve a durable and successful therapy. One of the critical challenges is protecting the transplanted cells against the immune systems of diabetic patients. In addition, the transplanted cells should be supported by enough nutrients and O_2_ for their survival until vasculogenesis occurs. Besides remaining viable, the islet should interact with the host microenvironment to maintain bioactivity since insulin secretion varies in response to changing glucose concentrations.

To address the mentioned challenges, engineering techniques are adopted to encapsulate *β*-cells in hydrogel-based biomaterials in various scales, from nano to macro. In the present review, the numerous approaches for fabricating hydrogel-based devices for *β*-cell encapsulation enhancing insulin secretion and survival of islet have been comprehensively reviewed.

Hydrogel-based devices present advanced formulations with superior characteristics, such as injectability, mimicking native islet ECM, enhancing oxygen and nutrient diffusion, and improving cells’ viability by immunosuppression. Recently, hydrogels designed for *β*-cells’ encapsulation have benefited from remarkable biomaterial synthesis and modification progress. Versatile methods of hydrogels’ synthesis techniques such as *in situ* formation, bioprinting, tissue decellularization, and self-assembling provide ample opportunity to encapsulate *β*-cells in a hydrogel-based carrier in various forms (e.g., injectable hydrogel, self-assembled structure, nano-/microparticles).

There is much interest in immunological hydrogels for *β*-cell delivery. These hydrogels are structurally and physicochemically modified by some modification techniques such as bulk/surface modification and incorporation by bioactive agent, immunosuppressant, or growth factors delivery system. Also, regarding the vital role of vasculogenesis, more advanced hydrogel could be multi-functionalized to stimulate angiogenesis or sustained O_2_ release in order to fully support the transplanted cells over time. Acute inflammation, hypoxia, cell necrosis, nutrient transport, and even mechanical failure can limit the efficacy of the encapsulation approach. Overcoming such challenges is the main focus of different studies. In the light of advances in nanoencapsulation devices, using nanoscale hydrogels considerably increases insulin and nutrient transport and safely protects cells from immune system elimination. High surface: volume ratio in the design of these devices considerably increases nutrient and transport. In the other point of view, better cell functionality is exhibited in nanoscale compared to micro- and macroscales. However, controlling the porosity and thickness of these devices is not fully achieved.

Although flexibility for transplantation site, retrieval, and reloading of cells and fibrosis formation are still big challenges needing to be tackled from a clinical perspective, it is undeniable that nanodevice fabrication for *β*-cell encapsulation is a moving forward and promising field in T1DM treatment.

Looking to the future, it seems that owing to remarkable progress in bio-manufacturing techniques such as 3D bioprinting, which can provide precise spatial geometry, mimic pancreatic islets microstructure, and embed vascular network inside the transplant, they could be a new practical and high efficient approach for islets encapsulation.

The next generation of biomaterials used for *β*-cell encapsulation should be formed by highly throughput fabrication techniques, especially nanofabrication, bioprinting, and highly efficient modification techniques such as coencapsulation of *β*-cells with other aiding cells (e.g., MSCs and vasculogenic cells). Actually, with the joint efforts of biomaterial scientists, the next generation of encapsulating material will be focused on biomimetic multi-functionalized hydrogels with superior performance for practical islets encapsulation along with their regeneration.

In summary, we envision that by overcoming the remaining challenges, scientist researchers will focus on developing hydrogel-based islet encapsulation in biomedical applications and commercializing products.

## References

[B1] Al-Enizi A. Zagho M. Elzatahry A. Elzatahry Ahmed. A. (2018). Polymer-Based Electrospun Nanofibers for Biomedical Applications. Nanomaterials 8 (4), 259–322. 10.3390/nano8040259 PMC5923589 29677145

[B2] Amani H. Arzaghi H. Bayandori M. Dezfuli A. S. Pazoki‐Toroudi H. Shafiee A. (2019). Controlling Cell Behavior through the Design of Biomaterial Surfaces: A Focus on Surface Modification Techniques. Adv. Mater. Inter. 6 (13), 1900572–1900630. 10.1002/admi.201900572

[B3] Bai X. Pei Q. Pu C. Chen Y. He S. Wang B. (2020). Multifunctional Islet Transplantation Hydrogel Encapsulating A20 High-Expressing Islets. Dddt Vol. 14, 4021–4027. 10.2147/DDDT.S273050 PMC7532915 33061306

[B4] Baidal D. A. Ricordi C. Berman D. M. Alvarez A. Padilla N. Ciancio G. (2017). Bioengineering of an Intraabdominal Endocrine Pancreas. N. Engl. J. Med. 376, 1887–1889. 10.1056/NEJMc1613959 28489987 PMC5572072

[B5] Bal T. Nazli C. Okcu A. Duruksu G. Karaöz E. Kizilel S. (2017). Mesenchymal Stem Cells and Ligand Incorporation in Biomimetic Poly(ethylene Glycol) Hydrogels Significantly Improve Insulin Secretion from Pancreatic Islets. J. Tissue Eng. Regen. Med. 11 (3), 694–703. 10.1002/term.1965 25393526

[B6] Banerjee J. Radvar E. Azevedo H. S. (2018). Self-Assembling Peptides and Their Application in Tissue Engineering and Regenerative Medicine. Peptides Proteins as Biomater. Tissue Regen. Repair, 245–281. 10.1016/B978-0-08-100803-4.00010-3

[B7] Barkai U. Weir G. C. Clark K. Ludwig B. Bornstein S. R. Brendel M. D. (2013). Enhanced Oxygen Supply Improves Islet Viability in a New Bioartificial Pancreas. Cel Transpl. 22, 1463–1476. 10.3727/096368912x657341 23043896

[B8] Bascones-Martinez A. Mattila R. Gomez-Font R. Meurman J. (2014). Immunomodulatory Drugs: Oral and Systemic Adverse Effects. Med. Oral 19 (1), e24–e31. 10.4317/medoral.19087 PMC3909428 23986016

[B9] Beenken-Rothkopf L. N. Karfeld-Sulzer L. S. Davis N. E. Forster R. Barron A. E. Fontaine M. J. (2013). The Incorporation of Extracellular Matrix Proteins in Protein Polymer Hydrogels to Improve Encapsulated Beta-Cell Function. Ann. Clin. Lab. Sci. 43 (2), 111–121. 23694784

[B10] Bellis S. L. (2012). Advantages of RGD Peptides for Directing Cell Association with Biomaterials. Biomaterials 32 (18), 4205–4210. 10.1016/j.biomaterials.2011.02.029.Advantages PMC3091033 21515168

[B11] Bochenek M. A. Veiseh O. Vegas A. J. McGarrigle J. J. Qi M. Marchese E. (2018). Alginate Encapsulation as Long-Term Immune Protection of Allogeneic Pancreatic Islet Cells Transplanted into the Omental Bursa of Macaques. Nat. Biomed. Eng. 2 (11), 810–821. 10.1038/s41551-018-0275-1 30873298 PMC6413527

[B12] Brissova M. Powers A. C. (2008). Revascularization of Transplanted Islets: Can it Be Improved?. Diabetes 57 (9), 2269–2271. 10.2337/db08-0814 18753672 PMC2518476

[B13] Brissova M. Shostak A. Shiota M. Wiebe P. O. Poffenberger G. Kantz J. (2006). Pancreatic Islet Production of Vascular Endothelial Growth Factor-A Is Essential for Islet Vascularization, Revascularization, and Function. Diabetes 55 (11), 2974–2985. 10.2337/db06-0690 17065333

[B14] Bünger C. M. Tiefenbach B. Jahnke A. Gerlach C. Freier T. Schmitz K. P. (2005). Deletion of the Tissue Response against Alginate-Pll Capsules by Temporary Release of Co-encapsulated Steroids. Biomaterials 26 (15), 2353–2360. 10.1016/j.biomaterials.2004.07.017 15585238

[B15] Calafiore R. Basta G. Luca G. Lemmi A. Montanucci M. P. Calabrese G. (2006). Microencapsulated Pancreatic Islet Allografts into Nonimmunosuppressed Patients with Type 1. Diabetes First Two Cases. Diabetes Care 29 (1), 1–2. 10.2337/diacare.29.01.06.dc05-1270 16373911

[B16] Camci-Unal G. Alemdar N. Annabi N. Khademhosseini A. (2013). Oxygen-Releasing Biomaterials for Tissue Engineering. Polym. Int. 62 (6), 843–848. 10.1002/pi.4502 23853426 PMC3708668

[B17] Cañibano-Hernández A. Saenz del Burgo L. Espona-Noguera A. Orive G. Hernández R. M. Ciriza J. (2019). Hyaluronic Acid Enhances Cell Survival of Encapsulated Insulin-Producing Cells in Alginate-Based Microcapsules. Int. J. Pharmaceutics 557 (December 2018), 192–198. 10.1016/j.ijpharm.2018.12.062 30597265

[B18] Cao Z. Wang X. (2014). The Endocrine Role between β Cells and Intra-islet Endothelial Cells [Review]. Endocr. J. 61 (7), 647–654. 10.1507/endocrj.ej14-0045 24681780

[B19] Carlsson P.-O. Palm F. Andersson A. Liss P. (2001). Markedly Decreased Oxygen Tension in Transplanted Rat Pancreatic Islets Irrespective of the Implantation Site. Diabetes 50 (3), 489–495. 10.2337/diabetes.50.3.489 11246867

[B20] Caserto J. S. Bowers D. T. Shariati K. Ma M. (2020). Biomaterial Applications in Islet Encapsulation and Transplantation. ACS Appl. Bio Mater. 3 (12), 8127–8135. 10.1021/acsabm.0c01235 35019595

[B21] Chang T. M. S. (1964). Semipermeable Microcapsules. Science 146, 524–525. 10.1126/science.146.3643.524 14190240

[B22] Chen S. Li R. Li X. Xie J. (2018). Electrospinning: An Enabling Nanotechnology Platform for Drug Delivery and Regenerative Medicine. Adv. Drug Deliv. Rev. 132, 188–213. 10.1016/j.addr.2018.05.001 29729295

[B23] Chen T. Yuan J. Duncanson S. Hibert M. L. Kodish B. C. Mylavaganam G. (2015). Alginate Encapsulant Incorporating CXCL12 Supports Long-Term Allo- and Xenoislet Transplantation without Systemic Immune Suppression. Am. J. Transplant. 15 (3), 618–627. 10.1111/ajt.13049 25693473

[B24] Cooper-Jones Brit. Ford Caitlyn. (2016), Islet Cell Replacement Therapy for Insulin-dependent Diabetes. CADTH Issues in Emerging Health Technologies, 1–9. Available at http://www.ncbi.nlm.nih.gov/pubmed/29369575. 29369575

[B25] Correia C. R. Ghasemzadeh-Hasankolaei M. Mano J. F. (2019). Cell Encapsulation in Liquified Compartments: Protocol Optimization and Challenges. PLoS ONE 14 (6), e0218045–12. 10.1371/journal.pone.0218045 31226115 PMC6588215

[B26] Crawford L. A. Guney M. A. Oh Y. A. Deyoung R. A. Valenzuela D. M. Murphy A. J. (2009). Connective Tissue Growth Factor (CTGF) Inactivation Leads to Defects in Islet Cell Lineage Allocation and β-Cell Proliferation during Embryogenesis. Mol. Endocrinol. (Baltimore, Md 23 (3), 324–336. 10.1210/me.2008-0045 PMC2654514 19131512

[B27] Crisóstomo J. Pereira A. M. Bidarra S. J. Gonçalves A. C. Granja P. L. Coelho J. F. (2019). ECM-enriched Alginate Hydrogels for Bioartificial Pancreas: An Ideal Niche to Improve Insulin Secretion and Diabetic Glucose Profile. J. Appl. Biomater. Funct. Mater. 17 (4), 228080001984892. 10.1177/2280800019848923 31623515

[B28] Daly A. C. Riley L. Segura T. Burdick J. A. (2020). Hydrogel Microparticles for Biomedical Applications. Nat. Rev. Mater. 5 (1), 20–43. 10.1038/s41578-019-0148-6 34123409 PMC8191408

[B29] De Miguel M. P. Fuentes-Julián S. Blázquez-Martínez A. Pascual C. Y. Aller M. A. Arias J. (2012). Immunosuppressive Properties of Mesenchymal Stem Cells: Advances and Applications. Curr. Mol. Med. 12 (5), 574–591. 10.2174/156652412800619950 22515979

[B30] Derakhshanfar S. Mbeleck R. Xu K. Zhang X. Zhong W. Xing M. (2018). 3D Bioprinting for Biomedical Devices and Tissue Engineering: A Review of Recent Trends and Advances. Bioactive Mater. 3 (2), 144–156. 10.1016/j.bioactmat.2017.11.008 PMC5935777 29744452

[B31] Desai T. Shea L. D. (2017). Advances in Islet Encapsulation Technologies. Nat. Rev. Drug Discov. 16 (5), 338–350. 10.1038/nrd.2016.232 28008169 PMC11286215

[B32] Dhamecha D. Movsas R. Sano U. Menon J. U. (2019a). Applications of Alginate Microspheres in Therapeutics Delivery and Cell Culture: Past, Present and Future. Int. J. Pharmaceutics 569 (May), 118627. 10.1016/j.ijpharm.2019.118627 PMC7073469 31421199

[B33] Diana J. Gahzarian L. Simoni Y. Lehuen A. (2011). Innate Immunity in Type 1 Diabetes. Discov. Med. 11 (61), 513–520. 21712017

[B34] Dimitrioglou N. Kanelli M. Papageorgiou E. Karatzas T. Hatziavramidis D. (2019). Paving the Way for Successful Islet Encapsulation. Drug Discov. Today 24 (3), 737–748. 10.1016/j.drudis.2019.01.020 30738185

[B35] Ding S. Serra C. A. Vandamme T. F. Yu W. Anton N. (2019). Double Emulsions Prepared by Two-step Emulsification: History, State-Of-The-Art and Perspective. J. Controlled Release 295 (December 2018), 31–49. 10.1016/j.jconrel.2018.12.037 30579983

[B36] Dinnyes A. Schnur A. Muenthaisong S. Bartenstein P. Burcez C. T. Burton N. (2020). Integration of Nano‐ and Biotechnology for Beta‐cell and Islet Transplantation in Type‐1 Diabetes Treatment. Cell Prolif 53 (5), 1–9. 10.1111/cpr.12785 PMC7260069 32339373

[B37] Dufrane D. Gianello P. (2012). Pig Islet for Xenotransplantation in Human: Structural and Physiological Compatibility for Human Clinical Application. Transplant. Rev. 26 (3), 183–188. 10.1016/j.trre.2011.07.004 22000658

[B38] Duin S. Schütz K. Ahlfeld T. Lehmann S. Lode A. Ludwig B. (2019). 3D Bioprinting of Functional Islets of Langerhans in an Alginate/Methylcellulose Hydrogel Blend. Adv. Healthc. Mater. 8 (7), 1801631–1801714. 10.1002/adhm.201801631 30835971

[B39] Elliott R. B. Escobar L. Tan P. L. J. Muzina M. Zwain S. Buchanan C. (2007). Live Encapsulated Porcine Islets from a Type 1 Diabetic Patient 9.5 Yr after Xenotransplantation. Xenotransplantation 14 (2), 157–161. 10.1111/j.1399-3089.2007.00384.x 17381690

[B40] Enck K. Tamburrini R. Deborah C. Gazia C. Jost A. Khalil F. (2021). Effect of Alginate Matrix Engineered to Mimic the Pancreatic Microenvironment on Encapsulated Islet Function. Biotechnol. Bioeng. 118 (3), 1177–1185. 10.1002/bit.27641 33270214 PMC8887826

[B41] Ernst A. U. Bowers D. T. Wang L.-H. Shariati K. Plesser M. D. Brown N. K. (2019). Nanotechnology in Cell Replacement Therapies for Type 1 Diabetes. Adv. Drug Deliv. Rev. 139, 116–138. 10.1016/j.addr.2019.01.013 30716349 PMC6677642

[B42] Espona-Noguera A. Ciriza J. Cañibano-Hernández A. Fernandez L. Ochoa I. Saenz Del Burgo L. (2018). Tunable Injectable Alginate-Based Hydrogel for Cell Therapy in Type 1 Diabetes Mellitus. Int. J. Biol. Macromolecules 107, 1261–1269. 10.1016/j.ijbiomac.2017.09.103 28962846

[B43] Espona-Noguera A. Ciriza J. Cañibano-Hernández A. Orive G. Hernández R. M. Saenz Del Burgo L. (2019). Review of Advanced Hydrogel-Based Cell Encapsulation Systems for Insulin Delivery in Type 1 Diabetes Mellitus. Pharmaceutics 11 (11), 597. 10.3390/pharmaceutics1111059 PMC6920807 31726670

[B44] Goncu B. Yucesan E. (2020). Microencapsulation for Clinical Applications and Transplantation by Using Different Alginates. Nano- Micro-Encapsulation - Tech. Appl. [Working Title], 1–12. 10.5772/intechopen.92134

[B45] Gorczynski R. M. (2005). CD200 and its Receptors as Targets for Immunoregulation. Curr. Opin. Investig. Drugs (London, England 6 (5), 483–488. 15912961

[B46] Guariguata L. Whiting D. R. Hambleton I. Beagley J. Linnenkamp U. Shaw J. E. (2014). Global Estimates of Diabetes Prevalence for 2013 and Projections for 2035. Diabetes Res. Clin. Pract. 103 (2), 137–149. 10.1016/j.diabres.2013.11.002 24630390

[B47] Gurlin R. E. Giraldo J. A. Latres E. (2021). 3D Bioprinting and Translation of Beta Cell Replacement Therapies for Type 1 Diabetes. Tissue Eng. B: Rev. 27, 238–252. 10.1089/ten.teb.2020.0192 32907514

[B48] Guziewicz N. Best A. Perez-Ramirez B. Kaplan D. L. (2011). Lyophilized Silk Fibroin Hydrogels for the Sustained Local Delivery of Therapeutic Monoclonal Antibodies. Biomaterials 32 (10), 2642–2650. 10.1016/j.biomaterials.2010.12.023 21216004 PMC3032024

[B49] Haque M. R. Lee D. Y. Ahn C.-H. Jeong J.-H. Byun Y. (2014). Local Co-delivery of Pancreatic Islets and Liposomal Clodronate Using Injectable Hydrogel to Prevent Acute Immune Reactions in a Type 1 Diabetes. Pharm. Res. 31 (9), 2453–2462. 10.1007/s11095-014-1340-4 24633416

[B50] Harrington S. Williams J. Rawal S. Ramachandran K. Stehno-Bittel L. (2017). Hyaluronic Acid/Collagen Hydrogel as an Alternative to Alginate for Long-Term Immunoprotected Islet Transplantation. Tissue Eng. A 23 (19–20), 1088–1099. 10.1089/ten.tea.2016.0477 PMC6112162 28142500

[B51] Hilbrands R. Huurman V. A. L. Gillard P. Velthuis J. H. L. De Waele M. Mathieu C. (2009). Differences in Baseline Lymphocyte Counts and Autoreactivity Are Associated with Differences in Outcome of Islet Cell Transplantation in Type 1 Diabetic Patients. Diabetes 58 (10), 2267–2276. 10.2337/db09-0160 19602536 PMC2750206

[B52] Hogan M. F. Hull R. L. (2017). The Islet Endothelial Cell: A Novel Contributor to Beta Cell Secretory Dysfunction in Diabetes. Diabetologia 60 (6), 952–959. 10.1007/s00125-017-4272-9 28396983 PMC5505567

[B53] Hu S. Martinez-Garcia F. D. Moeun B. N. Burgess J. K. Harmsen M. C. Hoesli C. (2021). An Immune Regulatory 3D-Printed Alginate-Pectin Construct for Immunoisolation of Insulin Producing β-cells. Mater. Sci. Eng. C 123 (December 2020), 112009. 10.1016/j.msec.2021.112009 33812628

[B54] Huang L. Xiang J. Cheng Y. Xiao L. Wang Q. Zhang Y. (2021). Regulation of Blood Glucose Using Islets Encapsulated in a Melanin-Modified Immune-Shielding Hydrogel. ACS Appl. Mater. Inter. 13 (11), 12877–12887. 10.1021/acsami.0c23010 33689267

[B55] Huang P. T. J. Shah D. K. Garcia J. A. Alexander G. C. Lim D.-J. Cui W. (2017). Encapsulation of Human Islets Using a Biomimetic Self-Assembled Nanomatrix Gel for Protection against Cellular Inflammatory Responces"*HHS Public Access* . ASC Biomater. Sci. Enj 3 (9), 2110–2119. 10.1021/acsbiomaterials.7b00261 PMC6615894 31289747

[B56] Huebsch N. (2019). Translational Mechanobiology: Designing Synthetic Hydrogel Matrices for Improved *In Vitro* Models and Cell-Based Therapies. Acta Biomater. 94, 97–111. 10.1016/j.actbio.2019.05.055 31129361

[B57] Hynes R. O. (2002). Integrins. Cell 110 (6), 673–687. 10.1016/s0092-8674(02)00971-6 12297042

[B58] Izeia L. Eufrasio-da-Silva T. Dolatshahi-Pirouz A. Ostrovidov S. Paolone G. Peppas N. A. (2020). Cell-Laden Alginate Hydrogels for the Treatment of Diabetes. Expert Opin. Drug Deliv. 17 (8), 1113–1118. 10.1080/17425247.2020.1778667 32515621

[B59] Jansen L. E. Amer L. D. Chen E. Y.-T. Nguyen T. V. Saleh L. S. Emrick T. (2018). Zwitterionic PEG-PC Hydrogels Modulate the Foreign Body Response in a Modulus-dependent Manner. Biomacromolecules 19 (7), 2880–2888. 10.1021/acs.biomac.8b00444 29698603 PMC6190668

[B60] Jeyhani M. Mak S. Y. Sammut S. Shum H. C. Hwang D. K. Tsai S. S. H. (2018). Controlled Electrospray Generation of Nonspherical Alginate Microparticles. ChemPhysChem 19 (16), 2113–2118. 10.1002/cphc.201701094 29228474

[B61] Jiang K. Chaimov D. Patel S. N. Liang J.-P. Wiggins S. C. Samojlik M. M. (2019). 3-D Physiomimetic Extracellular Matrix Hydrogels Provide a Supportive Microenvironment for Rodent and Human Islet Culture. Biomaterials 198, 37–48. 10.1016/j.biomaterials.2018.08.057 30224090 PMC6397100

[B62] João Paulo M. C. M. Leuckx G. Sterkendries P. Korf H. Bomfim-ferreira G. Overbergh L. (2016). Human Multipotent Adult Progenitor Cells Enhance Islet Function and Revascularisation when Co-transplanted as a Composite Pellet in a Mouse Model of Diabetes. Diabetologia 60, 134–142. 10.1007/s00125-016-4120-3 27704164 PMC6518081

[B63] Jourdan G. Dusseault J. Benhamou P. Y. Rosenberg L. Hallé J. P. (2011). Co-Encapsulation of Bioengineered IGF-II-Producing Cells and Pancreatic Islets: Effect on Beta-Cell Survival. Gene Ther. 18 (6), 539–545. 10.1038/gt.2010.166 21228884

[B64] Julier Z. Park A. J. Briquez P. S. Martino M. M. (2017). Promoting Tissue Regeneration by Modulating the Immune System. Acta Biomater. 53 (January), 13–28. 10.1016/j.actbio.2017.01.056 28119112

[B65] Kawakami Y. Iwata H. Gu Y. J. Miyamoto M. Murakami Y. Balamurugan A. N. (2001). Successful Subcutaneous Pancreatic Islet Transplantation Using an Angiogenic Growth Factor-Releasing Device. Pancreas 23 (4), 375–381. 10.1097/00006676-200111000-00007 11668206

[B66] Kengla C. Kidiyoor A. Murphy S. V. (2017). Bioprinting Complex 3D Tissue and Organs. Kidney Transplant. Bioeng. Regen. Kidney Transplant. Regenerative Med. Era, 957–971. 10.1016/B978-0-12-801734-0.00068-0

[B67] Kepsutlu B. Nazli C. Bal T. B. Kizilel S. (2014). Design of Bioartificial Pancreas with Functional Micro/Nano-Based Encapsulation of Islets. Cpb 15 (7), 590–608. 10.2174/1389201015666140915145709 25219869

[B68] Kerby A. Jones E. S. Jones P. M. King A. J., (2013). Co-Transplantation of Islets with Mesenchymal Stem Cells in Microcapsules Demonstrates Graft Outcome Can Be Improved in an Isolated-Graft Model of Islet Transplantation in Mice. Cytotherapy 15 (2), 192–200. 10.1016/j.jcyt.2012.10.018 23321331

[B69] Kharbikar B. N. Chendke G. S. Desai T. A. (2021). Modulating the Foreign Body Response of Implants for Diabetes Treatment. Adv. Drug Deliv. Rev. 174, 87–113. 10.1016/j.addr.2021.01.011 33484736 PMC8217111

[B70] Kim J. Kang K. Drogemuller C. J. Wallace G. G. Coates P. T. (2019b). Bioprinting an Artificial Pancreas for Type 1 Diabetes. Curr. Diab Rep. 19 (8), 53. 10.1007/s11892-019-1166-x 31273530

[B71] Kim J. Shim I. K. Hwang D. G. Lee Y. N. Lee M. Kim H. (2019a). 3D Cell Printing of Islet-Laden Pancreatic Tissue-Derived Extracellular Matrix Bioink Constructs for Enhancing Pancreatic Functions. J. Mater. Chem. B. 7 (10), 1773–1781. 10.1039/c8tb02787k 32254919

[B72] Klimek K. Ginalska G. (2020). Proteins and Peptides as Important Modifiers of the Polymer Scaffolds for Tissue Engineering Applications-A Review. Polymers 12 (4), 844. 10.3390/polym12040844 PMC7240665 32268607

[B73] Knobeloch T. Abadi S. E. M. Bruns J. Petrova Zustiak S. Kwon G. Kwon Guim. (2017). Injectable Polyethylene Glycol Hydrogel for Islet Encapsulation: an *In Vitro* and *In Vivo* Characterization. Biomed. Phys. Eng. Express 3, 035022. 10.1088/2057-1976/aa742b 29527325 PMC5842952

[B74] Krol S. Baronti W. Marchetti P. (2020). Nanoencapsulated Human Pancreatic Islets for β-cell Replacement in Type 1 Diabetes. Nanomedicine 15 (18), 1735–1738. 10.2217/nnm-2020-0166 32669019

[B75] Kryzstyniak Adam. Golab Karolina. Witkowski Piotr. Trzonkowski Piotr. (2014). Islet Cel Transpl. Incorporation Tregs 19 (6), 610–615. 10.1097/MOT.0000000000000130 PMC4270002 25304813

[B76] Kumagai-Braesch M. Jacobson S. Mori H. Jia X. Takahashi T. Wernerson A. (2013). The TheraCyte Device Protects against Islet Allograft Rejection in Immunized Hosts. Cel Transpl. 22 (7), 1137–1146. 10.3727/096368912X657486 23043940

[B77] Kwiatkowski A. J. Stewart J. M. Cho J. J. Avram D. Keselowsky B. G. (2020). Nano and Microparticle Emerging Strategies for Treatment of Autoimmune Diseases: Multiple Sclerosis and Type 1 Diabetes. Adv. Healthc. Mater. 9 (11), 2000164–2000211. 10.1002/adhm.202000164 PMC7588284 32519501

[B78] Laporte C. Tubbs E. Pierron M. Gallego A. Moisan A. Lamarche F. (2020). Improved Human Islets' Viability and Functionality with Mesenchymal Stem Cells and Arg-Gly-Asp Tripeptides Supplementation of Alginate Micro-encapsulated Islets *In Vitro* . Biochem. Biophysical Res. Commun. 528 (4), 650–657. 10.1016/j.bbrc.2020.05.107 32513541

[B79] Lau H. T. Yu M. Fontana A. Stoeckert C. J. (1996). Prevention of Islet Allograft Rejection with Engineered Myoblasts Expressing FasL in Mice. Science 273 (5271), 109–112. 10.1126/science.273.5271.109 8658177

[B80] Leberfinger A. N. Dinda S. Wu Y. Koduru S. V. Ozbolat V. Ravnic D. J. (2019). Bioprinting Functional Tissues. Acta Biomater. 95, 32–49. 10.1016/j.actbio.2019.01.009 30639351 PMC6625952

[B81] Lebish I. J. Moraski R. M. (1987). Mechanisms of Immunomodulation by Drugs. Toxicol. Pathol. 15 (3), 338–345. 10.1177/019262338701500312 3317771

[B82] Lee S. J. Lee J. B. Park Y.-W. Lee D. Y. (2018). 3D Bioprinting for Artificial Pancreas Organ. Adv. Exp. Med. Biol. 1064, 355–374. 10.1007/978-981-13-0445-3_21 30471043

[B83] Lew B. Kim I.-Y. Choi H. Kim K. (2018). Sustained Exenatide Delivery via Intracapsular Microspheres for Improved Survival and Function of Microencapsulated Porcine Islets. Drug Deliv. Transl. Res. 8 (3), 857–862. 10.1007/s13346-018-0484-x 29372538

[B84] Li S. Wang N. Guo X. Li J. Zhang T. Ren G. (2018). Fibroblast Growth Factor 21 Regulates Glucose Metabolism in Part by Reducing Renal Glucose Reabsorption. Biomed. Pharmacother. 108 (December), 355–366. 10.1016/j.biopha.2018.09.078 30227329

[B85] Liang J.-P. Accolla R. P. Soundirarajan M. Emerson A. Coronel M. M. Stabler C. L. 2021. “Engineering a Macroporous Oxygen-Generating Scaffold for Enhancing Islet Cell Transplantation within an Extrahepatic Site.” Acta Biomater. 10.1016/j.actbio.2021.05.028 34087442

[B86] Lim F. Sun A. (1980). Microencapsulated Islets as Bioartificial Endocrine Pancreas. Science 210 (4472), 908–910. 10.1126/science.6776628 6776628

[B87] Lin C. C. Raza A. Shih H. (2011). PEG Hydrogels Formed by Thiol-Ene Photo-Click Chemistry and Their Effect on the Formation and Recovery of Insulin-Secreting Cell Spheroids. Biomaterials 32 (36), 9685–9695. 10.1016/j.biomaterials.2011.08.083 21924490 PMC3195847

[B88] Lin Chien-chi. Anseth Kristi. S. (2009). Glucagon-Like Peptide-1 Functionalized PEG Hydrogels -Cells. Culture 4, 20–23. 10.1021/bm900420 PMC2745231 19586041

[B89] Lin Y.-J. Mi F.-L. Lin P.-Y. Miao Y.-B. Huang T. Chen K.-H. (2019). Strategies for Improving Diabetic Therapy via Alternative Administration Routes that Involve Stimuli-Responsive Insulin-Delivering Systems. Adv. Drug Deliv. Rev. 139, 71–82. 10.1016/j.addr.2018.12.001 30529306

[B90] Liu J. Liu S. Chen Y. Zhao X. Lu Y. Cheng J. (2015). Functionalized Self-Assembling Peptide Improves INS-1 β-Cell Function and Proliferation via the Integrin/FAK/ERK/Cyclin Pathway. Ijn 10, 3519–3531. 10.2147/IJN.S80502 25999715 PMC4436204

[B91] Liu J. Liu S. Zhang L. Cheng J. Lu Y. (2016b). Sustained Release of Hepatocyte Growth Factor by Cationic Self-Assembling Peptide/Heparin Hybrid Hydrogel Improves β-Cell Survival and Function through Modulating Inflammatory Response. Ijn Vol. 11, 4875–4890. 10.2147/IJN.S108921 PMC5042198 27729786

[B92] Liu J. M. H. Zhang J. Zhang X. Hlavaty K. A. Ricci C. F. Leonard J. N. (2016a). Transforming Growth Factor-Beta 1 Delivery from Microporous Scaffolds Decreases Inflammation Post-Implant and Enhances Function of Transplanted Islets. Biomaterials 80 (February), 11–19. 10.1016/j.biomaterials.2015.11.065D 26701143 PMC4706476

[B172] Liu T. Wang Y. Zhong W. Li B. Mequanint K. Luo G. (2018). Biomedical applications of layer-by-layer self-assembly. Adv. Healthcare Mater. 8, 18009. 10.1002/adhm.201800939 30511822

[B93] Liu Q. Chiu A. Wang L.-H. An D. Zhong M. Smink A. M. (2019). Zwitterionically Modified Alginates Mitigate Cellular Overgrowth for Cell Encapsulation. Nat. Commun. 10 (1), 1–14. 10.1038/s41467-019-13238-7 31748525 PMC6868136

[B95] Llacua L. A. Faas M. M. de Vos P. (2018). Extracellular Matrix Molecules and Their Potential Contribution to the Function of Transplanted Pancreatic Islets. Diabetologia 61 (6), 1261–1272. 10.1007/s00125-017-4524-8 29306997 PMC6449002

[B96] Lu J. Wang X. 2018, Biomimetic Self-Assembling Peptide Hydrogels for Tissue Engineering Applications. "Adv. Exp. Med. Biol. 1064,” 297–312. 10.1007/978-981-13-0445-3_18 30471040

[B97] Ludwig B. Reichel A. Steffen A. Zimerman B. Schally A. V. Block N. L. (2013). Transplantation of Human Islets without Immunosuppression. Proc. Natl. Acad. Sci. 110 (47), 19054–19058. 10.1073/pnas.1317561110 24167261 PMC3839710

[B98] Lugano R. Huang H. Dimberg A. (2017). “Vascular Endothelial Growth Factor Receptor (VEGFR).,” in Encyclopedia of Signaling Molecules. Editor Choi S. (New York, NY: Springer New York), 11–99. 10.1007/978-1-4614-6438-9_101914-1

[B99] Malektaj H. Imani R. Siadati M. H. (2021). Study of Injectable PNIPAAm Hydrogels Containing Niosomal Angiogenetic Drug Delivery System for Potential Cardiac Tissue Regeneration. Biomed. Mater. 16, 045031. 10.1088/1748-605X/abdef8 33482656

[B100] Mansour R. N. Soleimanifar F. Abazari M. F. Torabinejad S. Ardeshirylajimi A. Ghoraeian P. (2018). Collagen Coated Electrospun Polyethersulfon Nanofibers Improved Insulin Producing Cells Differentiation Potential of Human Induced Pluripotent Stem Cells. Artif. Cell Nanomedicine, Biotechnol. 46 (Suppl. 3), S734–S739. 10.1080/21691401.2018.1508031 30284483

[B101] Mayr J. Saldías C. Díaz Díaz D. (2018). Release of Small Bioactive Molecules from Physical Gels. Chem. Soc. Rev. 47, 1484–1515. 10.1039/c7cs00515f 29354818

[B102] Mitchell A. C. Briquez P. S. Hubbell J. A. Cochran J. R. (2016). Engineering Growth Factors for Regenerative Medicine Applications. Acta Biomater. 30 (January), 1–12. 10.1016/j.actbio.2015.11.007 26555377 PMC6067679

[B103] Mohamed-ahmed S. Fristad I. Suliman S. Mustafa K. Vindenes H. Idris S. B. 2018. “Adipose-Derived and Bone Marrow Mesenchymal Stem Cells: A Donor-Matched Comparison,”Stem Cel Res Ther. 9(1):168. 10.1186/s13287-018-0914-1 PMC6008936 29921311

[B104] Moini M. Schilsky M. L. Tichy Eric. M. (2015). Review on Immunosuppression in Liver Transplantation. Wjh 7 (10), 1355–1368. 10.4254/wjh.v7.i10.1355 26052381 PMC4450199

[B105] Montanucci P. Pescara T. Greco A. Leonardi G. Marini L. Basta G. (2020). Co‐microencapsulation of Human Umbilical Cord‐derived Mesenchymal Stem and Pancreatic Islet‐derived Insulin Producing Cells in Experimental Type 1 Diabetes. Diabetes Metab. Res. Rev. 37. 10.1002/dmrr.3372 32562342

[B106] Mridha A. R. Dargaville T. R. Dalton P. D. Carroll L. Morris M. B. Vaithilingam V. (2020). Prevascularized Retrievable Hybrid Implant to Enhance Function of Subcutaneous Encapsulated Islets. Tissue Eng. Part A, 1–35. 10.1089/ten.tea.2020.0179 33081600

[B107] Nagy N. Kaber G. Kratochvil M. J. Kuipers H. F. Ruppert S. M. Yadava K. 2020. “Sustained Release of IL-2 Using an Injectable Hydrogel Prevents Autoimmune Diabetes.” 10.1101/2020.03.15.993063 PMC7720893 33125521

[B108] Narayanaswamy R. Torchilin V. P. (2019). Hydrogels and Their Applications in Targeted Drug Delivery. Molecules 24 (3), 603. 10.3390/molecules24030603 PMC6384686 30744011

[B109] Nour S. Imani R. Chaudhry G. R. Sharifi A. M. (2020). Skin Wound Healing Assisted by Angiogenic Targeted Tissue Engineering: A Comprehensive Review of Bioengineered Approaches. J. Biomed. Mater. Res. 109, 453–478. 10.1002/jbm.a.37105 32985051

[B110] Ojaghi M. Soleimanifar F. Kazemi A. Ghollasi M. Soleimani M. Nasoohi N. (2019). Electrospun Poly‐ L ‐lactic Acid/polyvinyl Alcohol Nanofibers Improved Insulin‐producing Cell Differentiation Potential of Human Adipose‐derived Mesenchymal Stem Cells. J. Cel Biochem 120 (6), 9917–9926. 10.1002/jcb.28274 30548348

[B111] Omami M. McGarrigle J. J. Reedy M. Isa D. Ghani S. Marchese E. (2017). Islet Microencapsulation: Strategies and Clinical Status in Diabetes. Curr. Diab Rep. 17 (7), 1–7. 10.1007/s11892-017-0877-0 28523592

[B112] Ouyang L. Dan Y. Shao Z. Yang S. Yang C. Liu G. (2019). MMP-sensitive PEG H-ydrogel M-odified with RGD P-romotes bFGF, VEGF and EPC-mediated A-ngiogenesis. Exp. Ther. Med., 2933–2941. 10.3892/etm.2019.7885 31572536 PMC6755480

[B113] Pedraza E. Coronel M. M. Fraker C. A. Ricordi C. Stabler C. L. (2012). Preventing Hypoxia-Induced Cell Death in Beta Cells and Islets via Hydrolytically Activated, Oxygen-Generating Biomaterials. Proc. Natl. Acad. Sci. 109 (11), 4245–4250. 10.1073/pnas.1113560109 22371586 PMC3306668

[B114] Peloso A. Citro A. Zoro T. Cobianchi L. Kahler-Quesada A. Bianchi C. M. (2018). Regenerative Medicine and Diabetes: Targeting the Extracellular Matrix beyond the Stem Cell Approach and Encapsulation Technology. Front. Endocrinol. 9 (AUG), 1–9. 10.3389/fendo.2018.00445 PMC6127205 30233489

[B115] Pepper A. R. Gala-lopez B. Ziff O. James Shapiro A. M. (2013). Revascularization of Transplanted Pancreatic Islets and Role of the Transplantation Site Clin. Dev. Immunol., 2013, 352315. 10.1155/2013/352315 24106517 PMC3782812

[B116] Phelps E. A. Headen D. M. Taylor W. R. García P. M. Garcia A. J. (2013). Vasculogenic Bio-Synthetic Hydrogel for Enhancement of Pancreatic Islet Engraftment and Function in Type 1 Diabetes. Biomaterials 34 (19), 4602–4611. 10.1016/j.biomaterials.2013.03.012.Vasculogenic 23541111 PMC3628538

[B117] Phelps E. A. Templeman K. L. Thulé P. M. García A. J. (2015). Engineered VEGF-Releasing PEG-MAL Hydrogel for Pancreatic Islet Vascularization. Drug Deliv. Transl. Res. 5 (2), 125–136. 10.1007/s13346-013-0142-2 25787738 PMC4366610

[B118] Primavera R. Kevadiya B. D. Swaminathan G. Wilson R. J. De Pascale A. Decuzzi P. (2020). Emerging Nano- and Micro-technologies Used in the Treatment of Type-1 Diabetes. Nanomaterials 10 (4), 789. 10.3390/nano10040789 PMC7221526 32325974

[B119] Qi M. (2014). Transplantation of Encapsulated Pancreatic Islets as a Treatment for Patients with Type 1 Diabetes Mellitus. Adv. Med. 2014, 1–15. 10.1155/2014/429710 PMC4590955 26556410

[B120] Rackham C. L. Chagastelles P. C. Nardi N. B. Hauge-Evans A. C. Jones P. M. King A. J. F. (2011). Co-Transplantation of Mesenchymal Stem Cells Maintains Islet Organisation and Morphology in Mice. Diabetologia 54 (5), 1127–1135. 10.1007/s00125-011-2053-4 21267536

[B121] Ravnic D. J. Leberfinger A. N. Ozbolat I. T. (2017). Bioprinting and Cellular Therapies for Type 1 Diabetes. Trends Biotechnol. 35 (11), 1025–1034. 10.1016/j.tibtech.2017.07.006 28789815

[B122] Reys L. L. Vaithilingam V. SthijnsMireille M. M. J. P. E. Soares E. RademakersDe Bont T. Vries R. (2021). Fucoidan Hydrogels Significantly Alleviate Oxidative Stress and Enhance the Endocrine Function of Encapsulated Beta Cells. Adv. Funct. Mater., 2011205. 10.1002/adfm.202011205

[B123] Richardson T. Barner S. Candiello J. Kumta P. N. Banerjee. I. (2016). Capsule Stiffness Regulates the Efficiency of Pancreatic Differentiation of Human Embryonic Stem Cells. Acta Biomater. 35, 153–165. 10.1016/j.actbio.2016.02.025 26911881

[B124] Ruhela A. Kasinathan G. N. Rath S. N. Sasikala M. Sharma C. S. (2021). Electrospun Freestanding Hydrophobic Fabric as a Potential Polymer Semi-permeable Membrane for Islet Encapsulation. Mater. Sci. Eng. C 118 (August 2020), 111409. 10.1016/j.msec.2020.111409 33255012

[B125] Sabra G. Vermette P. (2013). A 3D Cell Culture System: Separation Distance between INS-1 Cell and Endothelial Cell Monolayers Co-cultured in Fibrin Influences INS-1 Cells Insulin Secretion. Biotechnol. Bioeng. 110 (2), 619–627. 10.1002/bit.24716 22949028

[B126] Samojlik M. M. Stabler C. L. 2021. “Designing Biomaterials for the Modulation of Allogeneic and Autoimmune Responses to Cellular Implants in Type 1 Diabetes.” Acta Biomater. 10.1016/j.actbio.2021.05.039 PMC9148663 34102338

[B127] Sanandiya N. D. Vasudevan J. Das R. Lim C. T. Fernandez J. G. (2019). Stimuli-Responsive Injectable Cellulose Thixogel for Cell Encapsulation. Int. J. Biol. Macromolecules 130, 1009–1017. 10.1016/j.ijbiomac.2019.02.135 30851322

[B128] Saroia J. Yanen W. Wei Q. Zhang K. Lu T. Zhang B. (2018). A Review on Biocompatibility Nature of Hydrogels with 3D Printing Techniques, Tissue Engineering Application and its Future Prospective. Bio-des. Manuf. 1 (4), 265–279. 10.1007/s42242-018-0029-7

[B129] Schaschkow A. Sigrist S. Mura C. Barthes J. Vrana N. E. Czuba E. (2020). Glycaemic Control in Diabetic Rats Treated with Islet Transplantation Using Plasma Combined with Hydroxypropylmethyl Cellulose Hydrogel. Acta Biomater. 102, 259–272. 10.1016/j.actbio.2019.11.047 31811957

[B130] Seo H. Son J. Park J.-K. (2020). Controlled 3D Co-culture of Beta Cells and Endothelial Cells in a Micropatterned Collagen Sheet for Reproducible Construction of an Improved Pancreatic Pseudo-tissue. APL Bioeng. 4 (4), 046103–046108. 10.1063/5.0023873 33195961 PMC7647615

[B131] Shapiro A. M. J. Lakey J. R. T. Ryan E. A. Korbutt G. S. toth E. Warnock G. L. (2000). Islet Transplantation in Seven Patients with Type 1 Diabetes Mellitus Using a Glucocorticoid-free Immunosuppressive Regimen. N. Engl. J. Med. 343, 230–238. 10.1056/NEJM200007273430401 10911004

[B132] Shi G.-J. Shi G.-R. Zhang J.-y. Zhang W.-j. Gao C.-y. Jiang Y.-p. (2018). Involvement of Growth Factors in Diabetes Mellitus and its Complications: A General Review. Biomed. Pharmacother. 101 (December 2017), 510–527. 10.1016/j.biopha.2018.02.105 29505922

[B133] Shrestha P. Regmi S. Jeong J.-H. (2020). Injectable Hydrogels for Islet Transplantation: A Concise Review. J. Pharm. Investig. 50 (1), 29–45. 10.1007/s40005-019-00433-3

[B134] Singh A. Peppas N. A. (2014). Hydrogels and Scaffolds for Immunomodulation. Adv. Mater. 26 (38), 6530–6541. 10.1002/adma.201402105 25155610 PMC4269549

[B135] Skrzypek K. Barrera Y. B. Groth T. Stamatialis D. (2018). Endothelial and Beta Cell Composite Aggregates for Improved Function of a Bioartificial Pancreas Encapsulation Device. Int. J. Artif. Organs 41, 152–159. 10.1177/0391398817752295 29546813 PMC6161570

[B136] Smink A. M. de Vos P. (2018). Therapeutic Strategies for Modulating the Extracellular Matrix to Improve Pancreatic Islet Function and Survival after Transplantation. Curr. Diab Rep. 18 (7). 10.1007/s11892-018-1014-4 PMC5960477 29779190

[B137] Soon-Shiong P. Heintz R. E. Merideth N. Yao Q. X. Yao Z. Zheng T. (1994). Insulin Independence in a Type 1 Diabetic Patient after Encapsulated Islet Transplantation. The Lancet 343 (8903), 950–951. 10.1016/s0140-6736(94)90067-1 7909011

[B138] Stabler C. L. Li Y. Stewart J. M. Keselowsky B. G. (2019). Engineering Immunomodulatory Biomaterials for Type 1 Diabetes. Physiol. Behav. 176 (1), 429–450. 10.1038/s41578-019-0112-5.Engineering PMC7332200 32617176

[B139] Stendahl J. C. Kaufman D. B. Stupp S. I. (2009). Extracellular Matrix in Pancreatic Islets: Relevance to Scaffold Design and Transplantation. Cel Transpl. 18 (1), 1–12. 10.3727/096368909788237195 PMC2724969 19476204

[B140] Stock A. A. Manzoli V. De Toni T. Abreu M. M. Poh Y.-C. Ye L. (2020). Conformal Coating of Stem Cell-Derived Islets for β Cell Replacement in Type 1 Diabetes. Stem Cel Rep. 14 (1), 91–104. 10.1016/j.stemcr.2019.11.004 PMC6962554 31839542

[B141] Su J. HuLowe B.-H. Lowe W. L. Kaufman D. B. Messersmith P. B. (2010). Anti-Inflammatory Peptide-Functionalized Hydrogels for Insulin-Secreting Cell Encapsulation. Biomaterials 31 (2), 308–314. 10.1016/j.biomaterials.2009.09.045 19782393 PMC2784009

[B142] Syed F. Bugliani M. Novelli M. Olimpico F. Suleiman M. Marselli L. (2018). Conformal Coating by Multilayer Nano-Encapsulation for the Protection of Human Pancreatic Islets: *In-Vitro* and *In-Vivo* Studies. Nanomedicine: Nanotechnology, Biol. Med. 14 (7), 2191–2203. 10.1016/j.nano.2018.06.013 30016718

[B143] Tan P. L. (2010). Company Profile: Tissue Regeneration for Diabetes and Neurological Diseases at Living Cell Technologies. Regenerative Med. 5, 181–187. 10.2217/rme.10.4 20210578

[B144] Taraghdari Z. B. Rana I. Mohabatpour F. (2019). A Review on Bioengineering Approaches to Insulin Delivery: A Pharmaceutical and Engineering Perspective. Macromolecular Biosci. 19 (4), 1–21. 10.1002/mabi.201800458 30614193

[B145] Toftdal M. S. Taebnia N. Kadumudi F. B. Andresen T. L. Frogne T. Winkel L. (2021). Oxygen Releasing Hydrogels for Beta Cell Assisted Therapy. Int. J. Pharmaceutics 602 (April), 120595. 10.1016/j.ijpharm.2021.120595 33892060

[B146] Townsend S. E. Gannon. M. (2019). Extracellular Matrix-Associated Factors Play Critical Roles in Regulating Pancreatic β-Cell Proliferation and Survival. Endocrinology 160 (8), 1885–1894. 10.1210/en.2019-00206 31271410 PMC6656423

[B147] Tuch B. E. Keogh G. W. Williams L. J. Wu W. Foster J. L. Vaithilingam V. (2009). Safety and Viability of Microencapsulated Human Islets Transplanted into Diabetic Humans. Diabetes Care 32 (10), 1887–1889. 10.2337/dc09-0744 19549731 PMC2752920

[B148] Tylek T. Blum C. Hrynevich A. Schlegelmilch K. Schilling T. Dalton P. D. (2020). Precisely Defined Fiber Scaffolds with 40 μm Porosity Induce Elongation Driven M2-like Polarization of Human Macrophages. Biofabrication 12 (2), 025007. 10.1088/1758-5090/ab5f4e 31805543

[B149] Veiseh O. C Doloff J. Ma M. Arturo J. V. Hei H. Bader A. R. (2015). Size- and Shape-dependent Foreugn Body Immune Response to Materials Implanted in Rodents and Non-human Primates. Nat. Mater. 14 (6), 643–651. 10.1038/nmat4290 25985456 PMC4477281

[B150] Wang D. Liu F. Zhu L. Lin P. Han F. Wang X. (2020a). FGF21 Alleviates Neuroinflammation Following Ischemic Stroke by Modulating the Temporal and Spatial Dynamics of Microglia/Macrophages. J. Neuroinflammation 17 (1), 1–17. 10.1186/s12974-020-01921-2 32867781 PMC7457364

[B151] Wang J. K. Cheam N. M. J. Irvine S. A. Tan N. S. Venkatraman S. Tay C. Y. (2020b). Interpenetrating Network of Alginate-Human Adipose Extracellular Matrix Hydrogel for Islet Cells Encapsulation. Macromol. Rapid Commun. 41 (21), 2000275–2000276. 10.1002/marc.202000275 32815257

[B152] Wang J. Li Y. Wang X. Wang J. Tian H. Zhao P. (2017a). Droplet Microfluidics for the Production of Microparticles and Nanoparticles. Micromachines 8 (1), 22–23. 10.3390/mi8010022

[B153] Wang K. Hao Y. Wang Y. Chen J. Mao L. Deng Y. (2019a). Functional Hydrogels and Their Application in Drug Delivery, Biosensors, and Tissue Engineering. Int. J. Polym. Sci. 2019, 1–14. 10.1155/2019/3160732

[B154] Wang L.-H. Ernst A. U. Flanders J. A. Liu W. Wang X. Datta A. K. (2021). An Inverse-Breathing Encapsulation System for Cell Delivery. Sci. Adv. 7 (20), eabd5835. 10.1126/sciadv.abd5835 33990318 PMC8121434

[B155] Wang S. Li J. Zhou Z. Zhou S. Hu Z. (2019b). Micro-/Nano-Scales Direct Cell Behavior on Biomaterial Surfaces. Molecules 24 (1), 1–13. 10.3390/molecules24010075 PMC6337445 30587800

[B156] Wang X. Ao Q. Tian X. Fan J. Tong H. Hou W. (2017b). Gelatin-Based Hydrogels for Organ 3D Bioprinting. Polymers 9 (9), 401. 10.3390/polym9090401 PMC6418925 30965706

[B157] Weaver J. D. Headen D. M. Aquart J. Johnson C. T. Shea L. D. Shea L. D. (2017). Vasculogenic Hydrogel Enhances Islet Survival, Engraftment, and Function in Leading Extrahepatic Sites. Sci. Adv. 3 (6), e1700184–10. 10.1126/sciadv.1700184 28630926 PMC5457148

[B158] Weaver J. D. Headen D. M. Coronel M. M. Hunckler M. D. Shirwan H. García A. J. (2018). Synthetic Poly(ethylene Glycol)‐based Microfluidic Islet Encapsulation Reduces Graft Volume for Delivery to Highly Vascularized and Retrievable Transplant Site. Am. J. Transpl. 19 (5), 1315–1327. 10.1111/ajt.15168 PMC6487074 30378751

[B159] Weber L. M. Anseth K. S. Anseth K. S. (2008). Hydrogel Encapsulation Environments Functionalized with Extracellular Matrix Interactions Increase Islet Insulin Secretion. Matrix Biol. 27 (8), 667–673. 10.1016/j.matbio.2008.08.001 18773957 PMC2631362

[B160] Wen Li. Zhang L. Ge X. Xu B. Zhang W. Qu L. 2018. Microfluidic Fabrication of Microparticles for Biomedical Applications. Chem. Soc. Rev. 47, 5646–5683. 10.1039/C7CS00263G 29999050 PMC6140344

[B161] Wirostko B. Wong T. Simó R. (2008). Vascular Endothelial Growth Factor and Diabetic Complications. Prog. Retin. Eye Res. 27 (6), 608–621. 10.1016/j.preteyeres.2008.09.002 18929676

[B162] Xu L. Guo Y. Huang Y. Xu Y. Lu Y. Wang Z. (2019). Hydrogel Materials for the Application of Islet Transplantation. J. Biomater. Appl. 33 (9), 1252–1264. 10.1177/0885328219831391 30791850

[B163] Yang J.-A. Yeom J. Hwang B. W. Hoffman A. S. Hahn S. K. (2014). In Situ-Forming Injectable Hydrogels for Regenerative Medicine. Prog. Polym. Sci. 39 (12), 1973–1986. 10.1016/j.progpolymsci.2014.07.006

[B164] Yap W. T. Salvay D. M. Silliman M. A. Zhang X. Bannon Z. G. Kaufman D. B. (2013). Collagen IV-Modified Scaffolds Improve Islet Survival and Function and Reduce Time to Euglycemia. Tissue Eng. Part A 19 (21–22), 2361–2372. 10.1089/ten.tea.2013.0033 23713524 PMC3807710

[B165] Yin N. Han Y. Xu H. Gao Y. Yi T. Yao J. (2016a). VEGF-conjugated Alginate Hydrogel Prompt Angiogenesis and Improve Pancreatic Islet Engraftment and Function in Type 1 Diabetes. Mater. Sci. Eng. C 59, 958–964. 10.1016/j.msec.2015.11.009 26652453

[B166] Youn W. Kim J. Y. Park J. Kim N. Choi H. Cho H. (2020). Single‐Cell Nanoencapsulation: From Passive to Active Shells. Adv. Mater. 32 (35), 1907001–1907017. 10.1002/adma.201907001 32255241

[B167] Zamboni F. Collins M. N. (2017). Cell Based Therapeutics in Type 1 Diabetes Mellitus. Int. J. Pharmaceutics 521 (1–2), 346–356. 10.1016/j.ijpharm.2017.02.063 28242376

[B168] Zhang J. Zhu Y. Song J. Xu T. Yang J. Du Y. (2019). Rapid and Long‐Term Glycemic Regulation with a Balanced Charged Immune‐Evasive Hydrogel in T1DM Mice. Adv. Funct. Mater. 29 (19), 1900140–1900149. 10.1002/adfm.201900140

[B169] Zhang L. Cao Z. Bai T. Carr L. Ella-Menye J.-R. Irvin C. (2013). Zwitterionic Hydrogels Implanted in Mice Resist the Foreign-Body Reaction. Nat. Biotechnol. 31 (6), 553–556. 10.1038/nbt.2580 23666011

[B170] Zhu Q. Lu C. Jiang X. Yao Q. Jiang X. Huang Z. (2019). Using Recombinant Human Collagen with Basic Fibroblast Growth Factor to Provide a Simulated Extracellular Matrix Microenvironment for the Revascularization and Attachment of Islets to the Transplantation Region. Front. Pharmacol. 10, 1536. 10.3389/fphar.2019.01536 31998133 PMC6965329

[B171] Zhu Y. Wang D. Yao X. Wang M. Zhao Y. Lu Y. (2020). Biomimetic Hybrid Scaffold of Electrospun Silk Fibroin and Pancreatic Decellularized Extracellular Matrix for Islet Survival. J. Biomater. Sci. Polym. Edition 32 (0), 151–165. 10.1080/09205063.2020.1818018 32867627

